# Contrasting structural complexity differentiate hunting strategy in an ambush apex predator

**DOI:** 10.1038/s41598-021-96908-1

**Published:** 2021-09-01

**Authors:** Milan Říha, Karl Ø. Gjelland, Vilém Děd, Antti P. Eloranta, Ruben Rabaneda-Bueno, Henrik Baktoft, Lukáš Vejřík, Ivana Vejříková, Vladislav Draštík, Marek Šmejkal, Michaela Holubová, Tomas Jůza, Carolyn Rosten, Zuzana Sajdlová, Finn Økland, Jiří Peterka

**Affiliations:** 1 Biology Centre of the Czech Academy of Sciences, Institute of Hydrobiology, České Budějovice, Czech Republic; 2 Norwegian Institute for Nature Research (NINA), Tromsö, Norway; 3 Department of Biological and Environmental Science, University of Jyväskylä, Jyväskylä, Finland; 4 National Institute of Aquatic Resources, Technical University of Denmark (DTU Aqua), Vejlsøvej 39, 8600 Silkeborg, Denmark; 5 Norwegian Institute for Nature Research (NINA), Trondheim, Norway

**Keywords:** Ecology, Behavioural ecology, Freshwater ecology, Stable isotope analysis

## Abstract

Structural complexity is known to influence prey behaviour, mortality and population structure, but the effects on predators have received less attention. We tested whether contrasting structural complexity in two newly colonised lakes (low structural complexity lake—LSC; high structural complexity—HSC) was associated with contrasting behaviour in an aquatic apex predator, Northern pike (*Esox lucius*; hereafter pike) present in the lakes. Behaviour of pike was studied with whole-lake acoustic telemetry tracking, supplemented by stable isotope analysis of pike prey utilization and survey fishing data on the prey fish community. Pike displayed increased activity, space use, individual growth as well as behavioural differentiation and spent more time in open waters in the LSC lake. Despite observed differences between lakes, stable isotopes analyses indicated a high dependency on littoral food sources in both lakes. We concluded that pike in the HSC lake displayed a behaviour consistent with a prevalent ambush predation behaviour, whereas the higher activity and larger space use in the LSC lake indicated a transition to more active search behaviour. It could lead to increased prey encounter and cause better growth in the LSC lake. Our study demonstrated how differences in structural complexity mediated prominent changes in the foraging behaviour of an apex predator, which in turn may have effects on the prey community.

## Introduction

The composition of biotic assemblages is heavily influenced by habitat heterogeneity arising from both abiotic and biotic components. Much focus has been given to the role that habitat complexity plays in structuring and functioning at the population level, both in aquatic and terrestrial ecosystems^1–3^. The presence of varied structures increases environmental heterogeneity and the range of available potential habitats^4^, and by providing shelter for prey or cover for predators structures have a crucial role in predator–prey interactions^5^. Physical structures, either biotic or abiotic, may also provide suitable substrate for primary producers, filtering organisms etc., and thus increase the food availability for predators^5^.

Submerged macrophytes are among the main structuring components in freshwater ecosystems. Macrophytes can substantially alter the behaviour of both prey and predators (e.g.^5–7^) via affecting predator–prey detection, encounter and catchability^8^. Some predators can alter their foraging behaviour and activity by switching between ambush and active pursuit^9–12^ depending on the presence/absence of macrophytes. This ability is species-specific and it has been documented in various groups of invertebrates^6,12^ and fish^9,11^, where it has implications for hunting success. Predators able to shift their foraging strategies may be able to maintain the total number of prey captures as the yield of their favoured foraging strategy is reduced^10–12^. However, a switch in activity and foraging behaviour to maintain prey consumption does not necessarily imply maintained predator growth and fitness, as higher activity costs might not be balanced by the energy gain, and thus be less efficient^6^. The most beneficial strategy will obviously be influenced by the rewards of the alternative strategies.

Besides the inherent ability of some species to modify foraging mode, recent research has revealed that there may be large individual variation in how these behavioural traits are expressed^13,14^. Such variability may be explained by sex-specific differences and individual variation in genetic and life-history traits^13,14^, some individuals may be more risk-prone than others, individual behaviour may be modified by social hierarchies^15^, or individuals may get more skilled and effective at one foraging tactic at the expense of other tactics^15,16^. Moreover, individual variation in foraging behaviour can also be linked with environmental complexity. Higher inter-individual dietary variation (directly connected with foraging behaviour) was found under low structural complexity, fostered by competition for macrophyte stands among individuals^17,18^. Although many predators may be able to change foraging mode, many of these mechanisms suggest individual foraging specialization to a specific foraging mode^15^.

Apex predators can potentially modify ecosystem structure and this ability is tightly linked with structural complexity in freshwater ecosystems^19^. Intraspecific variation in predator behaviour can determine prey abundance, community composition and trophic cascades^20^. Understanding apex predator behaviour and its variability in relation to structural complexity is therefore important to better understand their ability to cope with and/or elicit ecosystem changes, as well as the environmental drivers of phenotypic plasticity.

Research on behavioural responses of predators to changes in structural complexity has been carried out mainly at the species level in laboratory conditions or on small-scale experimental set-ups^6,9–12^. Such conditions may be unsuitable for large predators or they could lead to important behavioural traits being ruled out^21^. Data at the natural scale is sparse and fragmented, and our understanding of aquatic apex predator behaviour in natural conditions is still highly insufficient. The development of high-resolution tracking techniques^22^ now gives the opportunity to explore behaviour with unprecedented spatiotemporal resolution that could bring new insights into predator behaviour and their interaction with the environment^23^.

To test the effect of structural complexity on predator behaviour in natural conditions, we selected Northern pike (*Esox lucius*) as a model species. Pike is considered as an important model organism for identifying causes and consequences of phenotypic variation at the level of individuals and populations as well as for investigating community processes^24^. It is a freshwater apex predator typically associated with structurally complex habitats, often used as a classic example of a “sit-and-wait" ambush predator, and widely distributed in lakes and rivers across the Holarctic region^25,26^. Pike is a voracious forager with a wide variety of fish and other prey types and its introduction can have major impacts on fish species composition^25,27^. Recent research has revealed that pike can utilize a wider range of habitats, show active hunting in open water areas, and migrate many kilometres^28–30^. In some freshwater ecosystems, pike show behavioural types that differ in their level of activity and selection of habitat types^31^. Most seem to be strongly associated with structurally complex habitats, but this may also vary with season and ontogeny^32^. These findings suggest that pike have the capability for individual plasticity in habitat use and that differences in structural complexity may alter its foraging behaviour as a result of individual foraging specialization. On the other hand, some experimental studies indicate only minor effects of macrophyte density on pike behaviour^9^, and there is so far no evidence for how habitat complexity may alter the species’ behaviour in natural conditions.

We addressed the question of how pike behaviour changes with contrasting structural complexity by tracking pike with very high spatiotemporal resolution in two newly created post-mining lakes. The lakes have similar morphological and environmental parameters, but highly different submerged macrophyte structural complexity (low and high structural complexity, respectively LSC lake and HSC lake). Pike behaviour was investigated by examining habitat use and activity (telemetry), food availability in different habitats (gillnet and acoustic sampling of fish community), long-term diet (stable isotope analysis, SIA) and individual growth in tracked pike (scale analysis). We hypothesised that (1) space use (horizontal and vertical) will be inversely related to habitat complexity; (2) pike activity is inversely related to structural complexity; (3) the pelagic habitat and food resources will be more important in the lake with lower habitat complexity; (4) individual behavioural traits are consistent over time, but between-individual variation is higher in LSC lake; (5) growth is lower in the LSC lake, as activity costs are expected to be higher.

## Material and methods

### Study lakes

**Figure 1:**
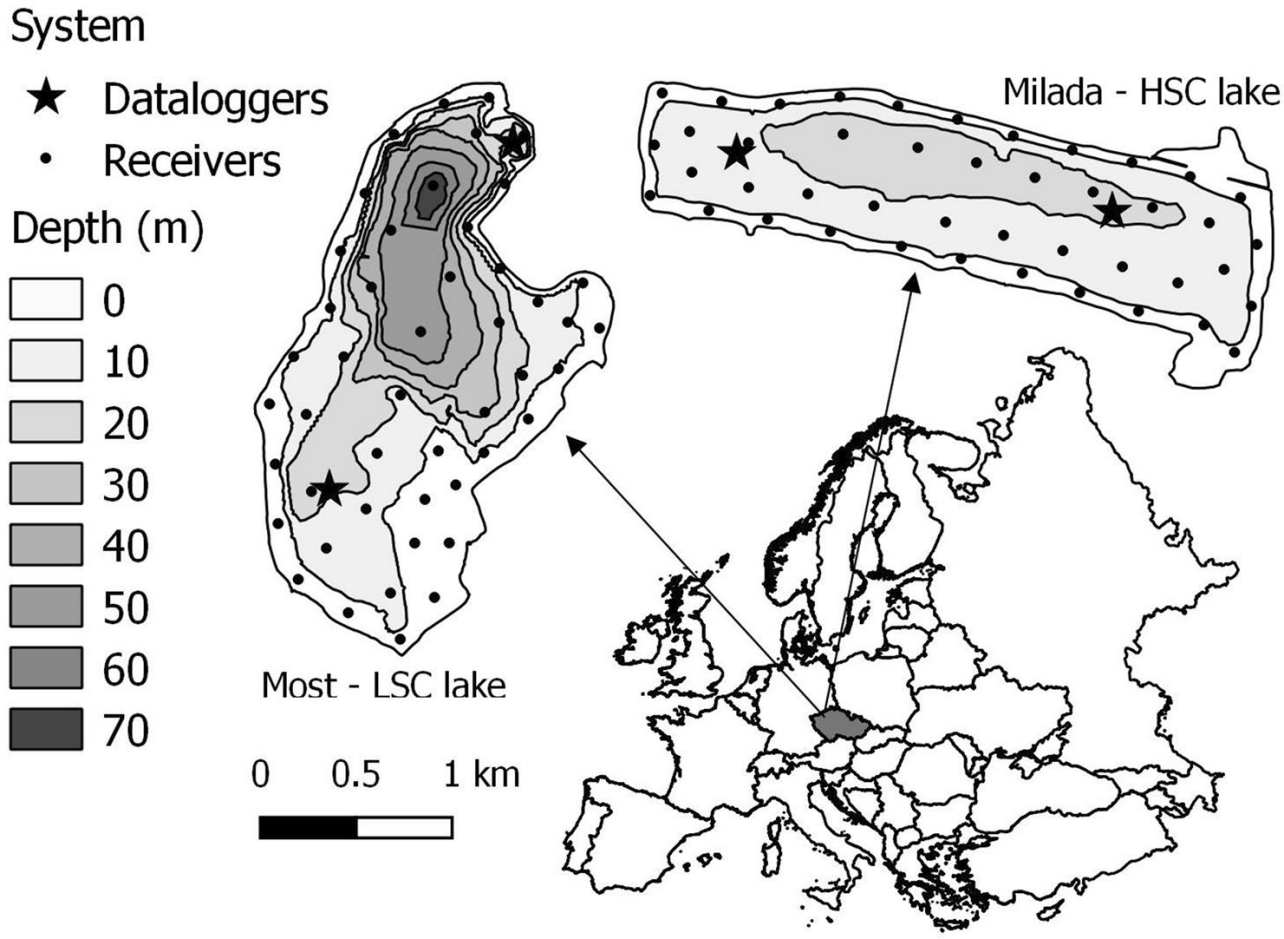
Locations and bathymetric maps of investigated lakes and positions of telemetry systems. Dots represent positions of each telemetry receiver and stars positions of temperature loggers. The map created using the Open Source QGIS version 3.18 (https://qgis.org/en/site/).

The study was conducted in two water bodies created after aquatic restorations of mining pits, Lakes Milada (2.5 km^2^, high structural complexity; 50° 39′ N, 13° 58′ E) and Most (3.1 km^2^, low structural complexity lake; 50° 32′ N, 13° 38′ E), in the Czech Republic (Fig. 1). Aquatic restoration took place from 2001 to 2010 in Milada and from 2008 to 2014 in Most. Both lakes are medium-sized (surface area = 252 and 311 hectares, respectively), relatively deep (*Z*mean = 16 and 22 m, *Z*max = 25 and 75 m), oligotrophic (mean summer total phosphorus < 10 and < 5 μg/L) and the Secchi depth varies between 3–10 m in Milada and 7–10 m in Most lake. The deeper Most has a well-oxygenated water column down to 50 m depth, whereas in Milada the profundal zone suffers from poor oxygen conditions below 20 m depth in summer^17^.

Macrophyte sampling was carried out prior to the study in September 2014 and May 2015^33^. Macrophytes were dense only in the HSC lake where they covered 60–91% of 0–12 m deep inshore areas. In the LSC lake, there was only a sparse macrophyte coverage of 0.1–1.6% at 0–3 m depth. Dominant macrophyte species in both lakes were *Potamogeton pectinatus*, *Myriophyllum spicatum* and *Chara* sp.

Both lakes had similar fish community compositions. Roach (*Rutilus rutilus*) and perch (*Perca fluviatilis*) were dominant in both lakes, while ruffe (*Gymnocephalus cernua*), tench (*Tinca tinca*), European catfish (*Silurus glanis*), Northern pike and pikeperch (*Sander lucioperca*) were less abundant^34^. In addition, pelagic planktivorous maraena whitefish (*Coregnus maraena*) were present in the LSC lake.

Stocking of predatory species including pike were performed in both lakes for biomanipulation purposes. Stocking of pike was performed after filling of the lakes as a biomanipulation measure to improve and maintain high water quality. In HSC lake, young-of the-year pike were stocked in 2005 and 1–2 year old pike in 2005, while in LSC lake over 1-year old pike were stocked in years 2011–2013 (Detail information in Supplementary material1 Tab. A1). Stocked pike came from various breeding ponds in the Central Bohemia region.

The pike populations were monitored long-term in both lakes and all captured pike were PIT tagged and released back in to the lake (more details are given in^35^). The population size estimate (based on recaptures) was around 220 individuals (size range 60–120 cm) in HSC lake in years 2014–2016. Low recaptures numbers did not allow making asimilar population size estimate in LSC lake. By comparing efforts and catches in both lakes, we estimated that population in LSC lake accounted for only 30–40% of that in HSC lake population (Vejřík L., unpublished data).

### Telemetry system

Two separate MAP positioning systems (Lotek Wireless Inc., Canada) were deployed in the HSC and LSC lakes to track tagged fish (see below). The systems consisted of 91 receivers (Lotek Wireless Inc., WHS3250; 44 receivers in HSC lake, and 47 receivers in the LSC lake) deployed in arrays with distances between the 3 nearest receivers ranging from 203 to 288 m (mean 251 ± 18 m) in the HSC lake and 191 to 341 m (mean 264 ± 33 m) in LSC lake (Fig. 1). The exact position of deployed receivers was measured using a high-precision GNSS-unit (Trimble Geo7x with a cm-precision RTK-service). Depth of receivers varied between 4.5 and 5.5 m. According to range testing done prior to the study, in these lakes (September 2014), such setting of receiver arrays should provide full coverage of both lakes under appropriate environmental conditions. Monitoring of the system accuracy was achieved by 20 stationary reference tags (10 tags in each lake; Lotek Wireless Inc., Canada, model MM-M-16-50-TP, burst rate 25 secs), located in 4 locations in each lake (2 open water locations at depths of 1, 5 and 13 m, and 2 nearshore locations at depths of 1 and 3 m). Further, testing of accuracy was performed by reference tags dragged below a boat after the deployment and before the final retrieval of the telemetry system (further information on positioning error are given in the Supplementary material 2). The systems were installed from April 2015 to March 2016 and the data was manually downloaded every two months. The period targeted in this study lasted from 27 May 2015 to 10 October 2015 to cover the summer period with the highest fish activity and development of macrophytes.

### Fish tagging

A total of 30 pike individuals (15 in each lake) were captured by electrofishing (23 individuals), long-lines (6 individuals) and angling (1 individual). Mean total body length/mass was 79 cm/4.13 kg for the HSC lake and 86 cm/4.15 kg for the LSC lake, respectively (more details in Table 1). After capture, pike were anaesthetized by 2-phenoxy-ethanol (SIGMA Chemical Co., USA, 0.7 ml L^−1^, mean time in anaesthetic bath was 3.75 min), measured, weighed and tagged. A 1–1.5 cm long incision was made on the ventral surface posterior to the pelvic girdle and the transmitter (Lotek Wireless Inc., MM-M-11-28-PM, 65 × 28 mm, mass in air of 13 g, including pressure and motion sensors, burst rate 25 s, tag weight ranged between 0.1 and 1.7% of fish body weigh; Table 1) was inserted through the incision and pushed forward into the body cavity. The incision was closed using two independent sutures. Mean surgery time was 2.8 min. In addition, scales for age determination and stable isotope analysis (see below) were taken during anaesthesia. All pike were released immediately after recovery from anaesthesia on the same location in each lake regardless of their capture location. Fish were captured and tagged between 5 and 10 May 2015.

**Table 1:** Description of tracked pike in both study lakes.

Lake	No. tagged/analyzed	Total length(cm)/Weight(kg)				Ratio of tag weight to body weigh (%)		
		Mean	SD	Min	Max	Mean	Min	Max
LSC	15/13	86.3/4.2	11.4/1.8	63.5/1.5	103.5/7.5	0.5	0.1	1.7
HSC	15/12	78.9/4.1	17.6/3.4	52/0.8	115/14.2	0.4	0.2	0.7

### Macrophyte sampling

To obtain an assessment of macrophyte assemblage and coverage, two SCUBA divers visually assessed macrophyte occurrence along 25 (HSC lake) and 26 (LSC lake) transects at the end of June and the beginning of September 2015. Sampling considered both aquatic plants as well as submerged dead terrestrial plants (only present in the LSC lake). Transects were situated from the shore to a depth of 12 m or deeper when macrophytes were present there. The coverage of each macrophyte species, the uncovered bottom area, the percentage composition of each species and maximum and minimum height of each macrophyte species were measured at 1 m depth intervals. The height of macrophytes was measured using a measuring tape. Dead flooded terrestrial plants were mostly European elder *Sambucus nigra* and thus categorized as a single group. Structural Complexity Index (hereafter referred to as SCI) in each lake was calculated to compare habitat complexity between lakes and its development during the study period. Calculation of SCI was based on information from the 25 and 26 (HSC and LSC lakes, respectively) scuba diver macrophyte assessment transects (see above). Species coverage and macrophyte height were both considered for calculation of the index. Species coverage and macrophyte height along each transect were georeferenced in a GIS-environment, and overlaid the bathymetric map for the lake. Height of macrophytes was discretized into 100 bins (from 0 to 200 cm by 2 cm) and coverage was discretized into 100 bins by 1%. The structural complexity index SCI was then defined as discretized height multiplied by discretized coverage. This gave a SCI range from 0 (no occurrence of macrophytes) to 10 000 (macrophyte coverage 100% and height ≥ 2 m).

### Temperature and oxygen measurement

To obtain abiotic parameters which can drive pike spatial distribution^36,37^, we monitored water temperature and oxygen concentration in both lakes. Water temperature was monitored using 60 data loggers (Onset, USA, HOBO Pendant temp/light 64 K). Data loggers were placed at two sites in each lake in order to cover east/west (HSC lake) and south/north (LSC lake) gradients (Fig. 1). At each site, data loggers were attached to a rope in 1 m intervals spanning from the surface to 13 m (14 data loggers) with one extra data logger located at a depth of 20 m. The rope was tied to a floating buoy anchored at 22 m depth. This setup ensured both dense coverage in depths of rapid temperature change and, with a measurement interval of 5 min, high spatiotemporal resolution of the temperature profile. Oxygen concentration was measured in each lake (once a month in the HSC lake, and once during the observed period in the LSC lake) by calibrated YSI 556 MPS probe (YSI Incorporated, USA). Measurements were performed close to the western (HSC lake) or northern (LSC lake) data logger station (Fig. 1).

### Fish community sampling

To obtain data of pike prey distribution, we performed spatially stratified fish community sampling by gillnet and hydroacoustic surveys. Gillnet surveys were conducted in September 2014 and 2015 at two localities in each lake in benthic habitats and one central locality in each lake for pelagic habitats, using 30 m long standard European multi-mesh gillnets^38^. At each locality in each lake, one series of three survey nets were set in the benthic and pelagic habitats at depths 0–3, 3–6, 6–9 and 9–18 m. In the deeper LSC lake, series of three survey nets were also set at depths 18–24, 24–30 and > 30 m. Benthic and pelagic gillnets were 1.5 m and 3 m high, respectively. Gillnets were set overnight, i.e. installed 2 h before sunset and lifted 2 h after sunrise^39^. Only catches of fish older than young-of-the-year were considered for this study. YOY fish were excluded as their density can be largely underestimated in gillnet surveys^40^. Moreover, Vejřík et al.^35^ found only prey fish larger than 10 cm in the stomachs of pike caught from both study lakes. Catches were expressed as catch per unit of effort measured as number of fish caught per 1000 m^2^ gillnet area per night (NPUE), and as kilogram fish 1000 m^−2^ night^−1^ (BPUE).

The acoustic surveys were performed both during the day and night, using a calibrated Simrad EK 60 echosounder operating at frequency of 120 kHz and following a pre-set zig-zag cruise track. The transducer was mounted 0.2 m below water surface, beaming vertically downwards. Recorded data were analysed using Sonar5-Pro software version 6.0.3^41^, using a Sv-threshold of − 62 dB (thresholded at 40 logR) and a target strength (TS) threshold of − 56 dB (corresponding to fish with an approximate total length of 4 cm^42^). Shoals were detected manually while fish tracking was used for individual fish. The following settings were used in the Sonar5-Pro auto-tracking tool to select fish tracks: minimum track length (MTL), maximum ping gap (MPG) and vertical range gating. MTL was set to 3 echoes, MPG was set to 1 and vertical gating to 0.15 m for the whole water column. Only areas deeper than 5 m were included in further analysis. The relative fish density was calculated for shoals (shoals ha^−1^) and individual fish (ind. ha^−1^) using the number of shoals or fish related to the surveyed area (calculated from the wedge formed by the distance, the range and the opening angle). The analysed water column was divided into depth layers (1 m thick, from 5 m below the surface to the bottom).

### Data processing

Positions of individual fish were calculated using a proprietary post-processing software UMAP v.1.4.3, based on multilateration of the time-difference-of-arrival (TDOA) of the acoustic signal received at different telemetry loggers (Lotek Wireless Inc., Newmarket, Ontario, Canada). Positions calculated using UMAP software can contain position duplicates and the use of TDOA for positioning implies large errors in a proportion of the position estimates^43^. Therefore, a position filter was applied in order to remove duplicate positions and positions with large errors. A detailed description of the filtering procedure is given in the SM1 (sec. Filtering of positions estimated by the U-MAP software). The position estimates (unfiltered and filtered) and depth profiles of each individual fish was visually inspected. If both horizontal and vertical locations became constant without latter movement, this individual was considered dead or expelling the tag (2 ind. in the LSC lake and 3 ind. in the HSC lake) and removed from further analyses.

The detection probability of transmitter detection and thus horizontal positioning changed over time and space due to underwater structures and thermal stratification. Numbers of position estimates for each individual ranged from 0 to 2884 per day (from 0 to 83% of possible number of position estimates). In order to reduce potential bias resulting from the varying positioning rate^44^, the individual data was regularized by calculating mean position for every 15-min interval (q-position hereafter). The q-position mean depth was calculated as the 15-min mean depth for each detected transmission with sensor depth reading. Since this did not require trilateration, the q-position time series could have a depth value without having a position estimate. This gave a maximum of 96 q-positions per day for each individual. If no position estimate was obtained within a 15-min interval, the q-position was interpolated between q-positions close in time if several conditions were fulfilled; (a) the time difference between the two q-positions used for interpolation (last before and first after the interval(s) to be interpolated) had to be shorter than 2 h, (b) the distance between these q-positions was less than 100 m, and (c) the depth difference between the q-positions was less than 2 m. Under such conditions, we assumed that the fish movement was limited between the q-positions used for interpolation (likely due to resting in an area with poor positioning coverage), and that linear interpolation in order to regularize the time series was therefore justified. For each position estimate, distance to bottom was calculated as the bottom depth at that position subtracted by the sensor depth. Distance to bottom for each q-position was calculated as the mean of each distance to bottom estimate within the 15-min interval. Individual and total yield of positions and calculated q-positions during the study period is given in Table 2.

**Table 2:** Yield of positions gathered by positioning systems in studied lakes (mean for all individuals, range among individuals and total sum of all gather positions) and calculated 15-min q-positions.

Lake	Raw positions				q-positions			
	Mean	Min	Max	Sum	Mean	Min	Max	Sum
LSC	68,745	6301	116,333	893,685	6429	1370	10,818	83,581
HSC	116,201	49,211	198,881	1,394,416	7568	3899	12,581	90,819

Depth of fish was measured by an internal tag sensor and transmitted together with an tag identification. Therefore, to get depth of fish, only detection of a single receiver was required (not three as for location of a horizontal position). Depth location was, therefore, obtained in much higher detail than fish horizontal location (fewer gaps). To synchronize depth and position with horizontal position, we calculated mean depth for the same 15-min intervals as we used for calculation of mean horizontal position from receiver detections at that interval.

Extent of horizontal area use was calculated using a 95% kernel utilization distribution (hereafter referred to as dH-KUD). dH-KUD was calculated for each day and individual separately and only for days with more than 12 daytime and 12 night-time q-positions. Night-time and daytime were defined as one hour after/prior to civil twilight periods^39^. Exact time of sunset and sunrise in each day was calculated using R-package “maptools”^45^ and dH-KUD was calculated using R-package “adehabitatHR”^46^. Parameters required for calculation of dH-KUD were set as follows:a simplified lake shape polygon was used as a boundary, a raster of a lake with a cell dimension of 10 × 10 m was used as a grid and the smoothing parameter h was set to value of 50. The extent of vertical movement was evaluated separately. Vertical space use (hereafter referred to as dV-KS) was calculated as a one-dimensional kernel estimate (95%) of Gaussian density function with the smoothing bandwidth parameter set to 0.4 fitted on distribution of utilized depths.

Activity of fish was calculated as horizontal swimming speed (expressed in body lengths per second, BL * s^−1^) and vertical swimming speed (depth change per second, m * s^−1^) between two consecutive q-positions.

To test the importance of an open water habitat for pike in both lakes, the proportion of time spent in open water was calculated (TOW). Each q-position was assigned to be either in the benthic (distance < 5 m from the bottom) or in the open water habitat (≥ 5 m from the bottom).

Daily water temperature and day length were abiotic factors considered to potentially drive pike behaviour in lakes^36,37,47^. Daily water temperature was calculated as the mean temperature from measurement of all data loggers at depths 0–3 m for each date during the study, separately for each lake. This parameter reflects both rapid daily and gradual seasonal temperature changes. Day length was calculated as time between sunrise and sunset.

### Stable isotopes and growth

Stable isotopes are widely used in studies of food-web structure and function, as well as individual specialization among consumer populations^48,49^. Here, we used stable carbon (δ^13^C) and nitrogen (δ^15^N) isotopes to estimate the relative reliance of individual pike on littoral carbon (food) resources (hereafter abbreviated as littoral reliance, *LR*). The LR estimates were calculated using the two-source isotopic mixing model described in Post^50^, where the δ^13^C value of a consumer (measured from the outermost annual ring of pike scales) is compared to those of littoral and pelagic isotopic end-members. We used δ^13^C values of littoral macrophytes and benthic algae, and pelagic particulate organic matter (POM) as the littoral and pelagic isotopic end-members, respectively, because some pike individuals and herbivorous prey fishes had considerably higher δ^13^C values than littoral benthic invertebrates. For more details of sample collection and preparation for stable isotope analyses, see the previous studies of predatory^35^ and generalist^17^ fishes in the two study lakes.

Age determination and growth calculation for each individual were conducted using scale reading. Three scales were read for each individual, results were then averaged and used for back-calculation of size-at-age of each individual using the Fraser–Lee Equation^51^. Only the body increment during the year prior to tagging was used to test for the correlation of individual growth with the rates of horizontal and vertical movement, since this was the growth increment most relevant to the activity and body size during the study year.

### Statistical analyses

#### Behavioural traits and use of the pelagic zone

The response variables analyzed were horizontal area use (dH-KUD), vertical space use (dV-KS), daily mean depth (hypothesis i); horizontal activity, vertical activity (hypothesis ii); and time spent in open water (TOW, hypothesis iii); all of which could be associated with either a foraging or cover context. To test whether individuals from both lakes exhibited consistent behavioural differences across time we fitted a series of random-effects models and selected the best one based on Akaike Information Criterion (AIC, hypothesis iv). These models included a different set of covariates previously selected through Random Forest (hereafter RF) regression analysis^52^ using the R package *randomForest*^ 53^. RF is suitable for the identification of relevant interactions between variables with low to negligible effects in isolation (further description in SM1, sec. Identifying interactions with Random Forest), and thus it would allow us to constraint both the number and potential interactions between them.

For the analysis of dH-KUD, dV-KS, mean depth horizontal activity and vertical activity we first fitted full random-intercepts and random-slopes mixed-effects models (LMMs)^54,55^ with Gaussian error distribution using the package *nlme*^54^. These models all include the same relevant predictors and interactions from the RF analysis, which yielded quite consistent results between the analyzed variables. Fish body length and daily water temperature were included, prior transformation by z-score standardization^56^, as continuous covariates in the model. The factor Lake was used as a proxy of structural habitat complexity (SHC). To account for the individual variation in repeated-measures (see SM1 for more details), we added the unique identifier (tag identification number) as a random intercept. We included the time × Lake interaction with time both as a random slope effect and a fixed effect to model mean between-lake temporal trends. Time as a fixed effect represents the overall effect of time among individuals in the two lakes while time as a random intercept measures the variance of the temporal effects in response across individuals (i.e., repeatability)^57^. This random-effects structure was also always selected in alternative analyses using log-likelihood ratio tests^58,59^.

TOW was analysed using zero–one beta inflated models^60,61^ fitted with the function gamlss() in R package *gamlss*^62^ by specifying a BEINF distribution. GAMLSS models are a particular type of GAM for Location, Scale and Shape which allow mixed distributions of continuous observations in the range 0–1 and discrete values at 0 and 1 often representing probabilities ruled by different processes^63^. Models also allowed evaluation of the probabilities at the extremes. We assumed that the probability that the time spent in open water is zero (p_0_) is associated with a behavior involving inexistent use of the pelagic habitat. On the other hand, the probability at one (p_1_) would imply that fish fully use the pelagic habitat. In the range between 0 and 1, TOW is modelled as a i.i.d. Gaussian variable indicating variable levels of pelagic habitat use. These probabilities are therefore governed by different processes reflecting variations in behavior and are integrated in a single model through four distribution parameters (μ, σ, ν, τ) (see SM1 for more details on GAMLSS model parametrization; sec. GAMLSS model of pelagic habitat use). Each component was modelled as a function of the between-lake temporal trends (time × Lake interaction) controlling for the effect of dH-KUD and dV-KS (as well as body length) on TOW to account for the fact that large differences in vertical and horizontal range influence pelagic habitat use. Each continuous covariate was initially fitted using a penalized P-spline smoothing function, denoted by pb() in R syntax, which automatically selects the smoothing parameter (indicative of the degree of smoothness or complexity of the fitted curve). In the final models not all distribution parameters were necessarily modelled using an additive term as it depended on decisions made during the model selection procedure (see SM1 for further details on GAMLSS model parametrization, sec. GAMLSS model of pelagic habitat use).

To account for past error residual correlations and prevent pseudo-replication^55^, all models were fitted using an autocorrelation structure. This allowed us to further assess the differences in temporal trends by correctly partitioning inter- and intra-individual sources of variance^54,55^ whilst accounting for slowly fluctuating trait values or behavioural lags about those timelines. We selected the best autocorrelation structure by comparing models according to AIC^64,65^ and by previously visualizing ACF and PACF residuals to determine the starting lag value (*rho*)^66^. Along with the individual identity, time was included as a continuous variable to control for the correlation of residuals between any given days.

In a second step, once a full model was fitted, we created sets of candidate models with a different number of predictors and ranked them according to their AICc values (i.e., AIC with finite-size correction^67^, lower is better) and weights (higher is better), and finally compared. Here, the importance of the interaction terms detected in the RF analysis was further tested using likelihood ratio tests (for more details see SM1). The ranking and selection of LMMs was conducted using the R package *AICcmodavg*^68^. For GAMLSS models we used the different functions in the *gamlss* package to conduct a stepwise selection of appropriate terms for each distribution parameter (μ, σ, ν, τ) (see SM1 for a detailed description of the GAMLSS selection procedure utilized, sec. GAMLSS model of pelagic habitat use).

As a last step, models were re-fitted using restricted maximum likelihood (REML) and variance components assessed with the package *MuMIn*^69^. We calculated between- and within-individual variance components and estimated the repeatability (*R*) index, both on data from the two lakes and on subsets of data for each lake, to evaluate qualitative differences of repeatable patterns in temporal trend lines. Repeatability was calculated as:

$$\frac{{{\text{V}}_{{{\text{tag}}\_{\text{ID}}}} }}{{\left( {{\text{V}}_{{{\text{tag}}\_{\text{ID}}}} + {\text{V}}_{{\text{e}}} } \right)}},$$

where (V_tag_ID_ + V_e_) is the total phenotypic variance (V_p_), resulting from adding the between-individuals variance (V_tag_ID_) to the within-individual (residual) variance (V_e_). A high *R* may indicate high between- and/or low within- individual variability in the outcome values (see SM1 for further details on repeatability index). As a separate measure of repeatability, we compared the individual ranks in horizontal space use between June-July, June–August, and June–September using the Spearman rank correlation test^70^. The same procedure was done for swimming activity.

#### Stable isotopes

We ran linear models to test if the trophic niche of pike was related to individuals’ behaviour (open-water use as a proxy), structural habitat complexity (SHC; lake factor as a proxy) or body length (i.e., total length). Prior to modelling, open-water use was logit-transformed and continuous explanatory variables were scaled (see above).

#### Growth

We used linear regression to find differences in individual growth (hypothesis v) in the previous season (log-transform of body increment in year 2014; dependent variable) between lakes and whether they were determined by the space use (mean daily dH-KUD and dV-KS), body length and age of fish (explanatory variables).

### Ethics approval and consent to participate

This study complied with and was approved by the Animal Welfare Committee of the Biology Centre CAS (45/2014) according to § 16a of the Act No. 246/1992 Coll., on the protection of animals against cruelty, as amended. The study was carried out in compliance with the ARRIVE guidelines.

### Consent for publication

This manuscript presents work that has not been published and is not under consideration for publication elsewhere. All authors involved in the manuscript have agreed to be listed and contributed to the research reported.

## Results

### Seasonal development of lakes environment

**Figure 2:**
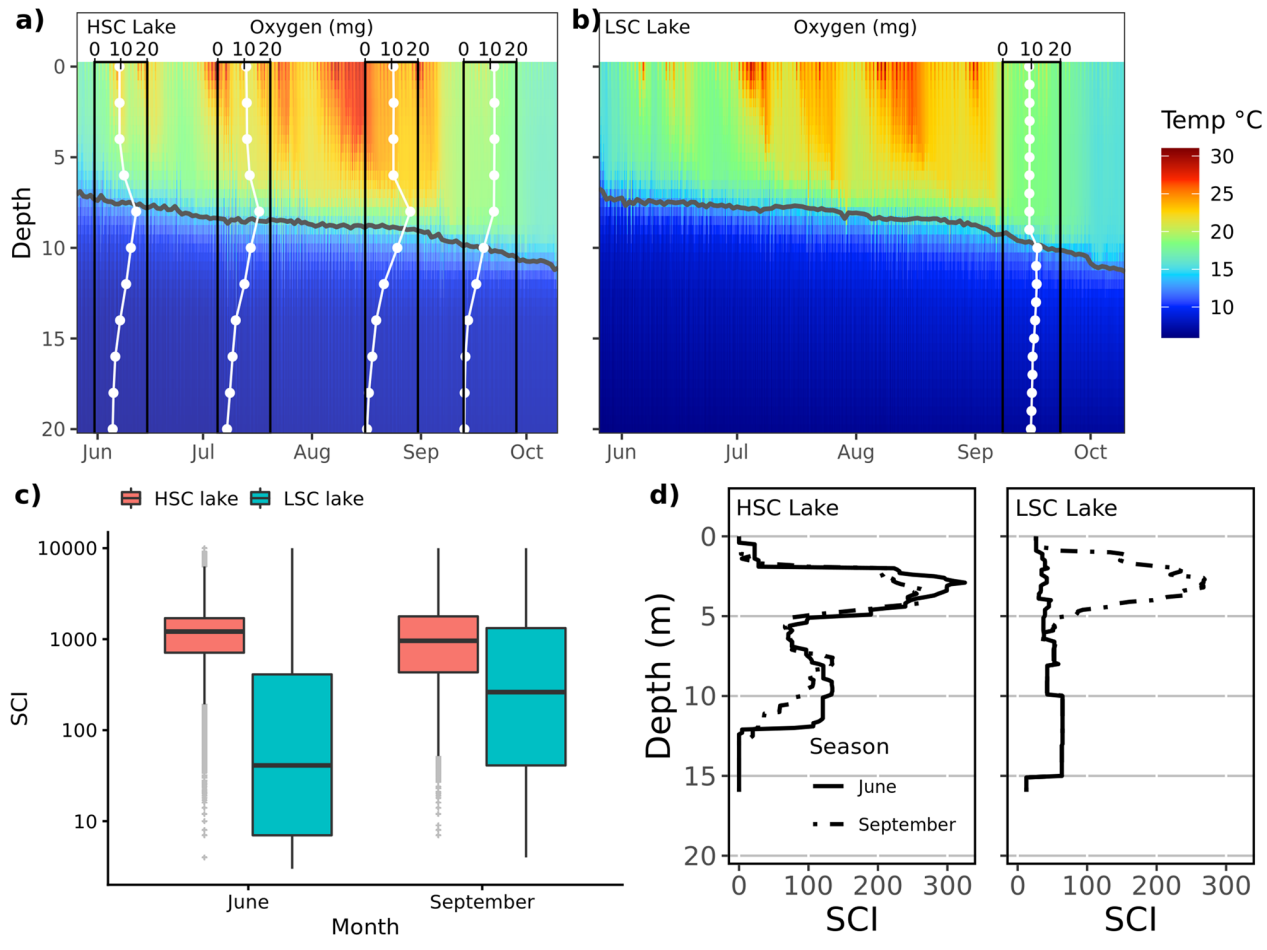
Temperature and oxygen stratification of water column at HSC (**a**) and LSC lakes (**b**), (**c**) distribution of SCI in SCI-rasters covering 0–15 m depth (SCI) in both lakes and (**d**) vertical distribution of the Structural Complexity Index (SCI) in both lakes. In (**c**) each point represents SCI value for 1 m^2^ of bottom. The bar in the middle of the box shows the median, lower and upper hinges of the box correspond to the 25th and 75th percentiles. The lower and upper whisker extends from the hinges to the smallest and largest value, respectively, no further than 1.5 * IQR from the hinge (where IQR is the inter-quartile range, i.e. the distance between the 25th and 75th percentiles). Note the logarithmic scale of the y-axis. In (**d**) lines show mean SCI on depth profile, given separately for each macrophyte sampling session.

Both lakes were thermally stratified with the thermocline gradually declining from 6–7 to 9–10 m during the study period (Fig. 2a,b). Oxygen concentration declined below the thermocline during the season in the HSC lake, to such an extent that the water was anoxic from 14 m depth and deeper in September (Fig. 2a), whereas oxygen concentrations were similar (around 10 mg/L) throughout the water column in the LSC lake at end of September (Fig. 2b). As expected the mean Structural Complexity Index was significantly higher in the HSC lake than in the LSC lake (*P* < 0.001; tested using permutation inference, SM1 sec. Testing of Structural Complexity Index differences between lakes), but this difference became much less in September as the Structural Complexity Index was relatively unchanged in the HSC lake, but greatly increased in the LSC lake (*P* < 0.001 for the lake by macrophyte period interaction; Fig. 2c,d and areal distributions of SCI is given in SM Fig. S7).

**Figure 3:**
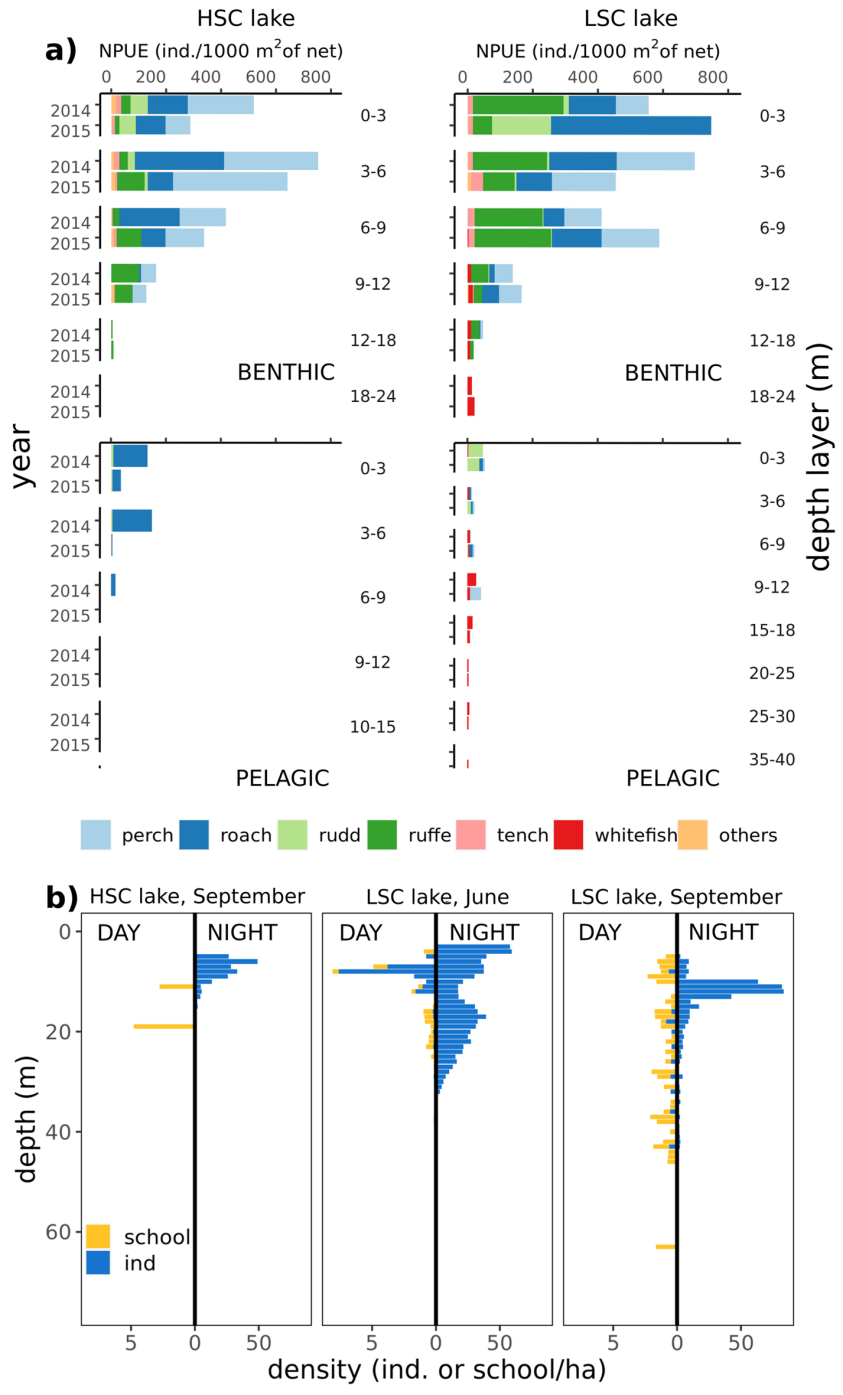
(**a**) Gillnet abundance of fish stock in both lakes, given separately for each sampled depth layer and separately for bentic and pelagic habitats in one year prior to study (2014) and during study period (2015). (**b**) vertical distribution of open water hydroacoustic densities separately in both lakes and diel periods.

Gillnet fish densities in terms of both number and biomass were higher in the benthic habitats than in the pelagic habitats in both lakes (Fig. 3a). In benthic habitats, gillnet fish densities and species composition were similar in both lakes, following the same vertical pattern with a steep decrease with depth and low density of fish below 12 m (Fig. 3a). Overall pelagic gillnet fish densities were slightly higher in the LSC lake, with whitefish present in low numbers even in the largest sampled depth layers (30–35 m). Whitefish were not detected in the HSC lake and almost exclusively only roach was captured in the pelagic zone in depths down to 9 m (Fig. 3a).

Hydroacoustic data showed almost no fish (school or single) in the HSC lake in September during the daytime and only low abundance of fish at night (Fig. 3b). In the LSC lake, low densities of both schools and single fish were detected in open water during the daytime in June and September and the number of single fish considerably increased at night in the habitat during both months sampled (Fig. 3b).

### Horizontal and vertical space use

The final models for horizontal and vertical utilization distributions, daily mean depth and horizontal and vertical activity are summarized in Table 3. Model selection tables are shown in Tables A2–A6 (Supplementary material).

**Table 3:** Final linear mixed-effects models analysing pike behavioural traits in low (LSC) and high (HSC) structurally complex lakes.

	dH-KUD log(ha)	dV-KS log(m)	Horizontal activity sqrt(m/s)	Vertical activity sqrt(m/s)	Depth log(m)
(Intercept)	0.77*** [0.625–0.92]	1.54*** [1.27–1.80]	0.045*** [0.035–0.06]	0.005*** [0.004–0.006]	1.47*** [1.20–1.75]
Time × lake	− 0.003* [− 0.005–2 × 10^–05^]	0.002 [− 0.002–0.005]	− 2.00** [− 4.00–0.40] × 10^–04^	1.00 [− 1.00–3.00] × 10^–05^	0.002 [− 0.002–0.006]
Time	0.002* [− 3 × 10^–04^–0.004]	− 0.001 [− 0.003–0.002]	0.30 [− 1.00–1.00] × 10^–04^	0.00 [− 1.00–1.00] × 10^–05^	0.004*** [0.001**–**0.007]
Lake	0.39*** [0.18–0.60	0.11 [− 0.08–0.30]	0.03*** [0.02–0.05]	0.001 [− 0.001–0.002]	− 0.21 [− 0.60**–**0.18]
Body length × lake	0.24*** [0.11–0.37]				
Body length	0.14*** [0.06–0.21]		0.006** [0.001**–**0.01]		0.19** [0.05**–**0.34]
Water temperature × body length	0.02* [− 0.002–0.04]				
Water temperature	− 0.03** [− 0.05–0.005]	− 0.02*** [− 0.03–0.005]	− 0.003*** [− 0.004–0.001]	− 2.00*** [− 3.00–1.00] × 10^–04^	0.05** [0.004–0.10]
**Random effects**					
σ^2^_e_	0.08^†^ [0.070–0.085]	0.18^†^ [0.17–0.20]	3.81^†^ [3.45–4.21] × 10^–04^	3.66^†^ [3.37–3.97] × 10^–06^	0.27^†^ [0.24–0.31]
τ_00_ _tag_id_	0.06*** [0.028–0.122]	0.04*** [0.014–0.12]	2.84*** [1.36–5.95] × 10^–04^	1.51*** [0.59–3.83] × 10^–06^	0.18*** [0.08–0.43]
τ_11_ _time_	1.02*** [0.50–2.08] × 10^–05^	1.64*** [0.76–3.53] × 10^–05^	3.56*** [1.63–7.80] × 10^–08^	4.04*** [1.88–8.65] × 10^–10^	1.96*** [0.72–5.34] × 10^–05^
ρ_01_ _tag_id.time_	− 0.82*** [− 0.93–0.58]	− 0.75*** [− 0.91–0.37]	− 0.73*** [− 0.89–0.38]	− 0.60** [− 0.84–0.14]	− 0.64** [− 0.87–0.16]
ρ _AR(1)_	0.72*** [0.65–0.77]	0.59*** [0.46–0.69]	0.73*** [0.66–0.79]	0.54*** [0.42–0.64]	0.78*** [0.72–0.82]
ρ _MA(1)_	− 0.26*** [− 0.34–0.18]	− 0.21*** [− 0.35–0.07]	− 0.24*** [− 0.33–0.15]	− 0.11*** [− 0.25–0.03]	− 0.12*** [− 0.21–0.04]
*R*	0.43	0.19	0.43	0.29	0.40
*R*_*LSC*_* | R*_HSC_	0.49 | 0.31	0.15 | 0.18	0.36 | 0.30	7.25 × 10^–05^ | 0.51	0.40 | 0.41
R^2^_m_/R^2^_c_	0.43/0.60	0.07/0.29	0.23/0.47	0.08/0.40	0.15/0.43

The variables analysed were both horizontal (dH-KUD) and vertical (dV-KS) utilization distributions, horizontal and vertical activity, and mean daily depth. All variables were modelled by fitting an ARMA autocorrelation structure of order (*p* = 1, q = 1). Data show β standardized estimates (mean-centred and scaled by 2 s.d.) and 95% confidence intervals; the response variable remains untransformed. LSC lake was set as the reference level. Random effects: *σ*^*2*^_*e*_, residual (within-individual) error variance; *τ*_*00*_ _*tag_id*,_ random intercept variance (i.e., variation between individual intercepts and average intercept); *τ*_*11*_ _*tag_id.time*_*,* random slope variance (i.e., variation in individual temporal slopes across days)*; ρ*_*01*_ _*tag_id*_*,* random slope-intercept correlation (i.e., correlation between the individual random intercepts and slopes); *R*, adjusted repeatability computed from fitted LMMs measuring the proportion of intra and/or inter- individual variation over time; *R *_*SHC*_*, R*_LSC_, adjusted repeatability computed for each lake dataset separately (see main text for more details). Significance values of random-effects parameters were computed using likelihood ratio tests between each two nested models varying only in their random-effects structure (only *p*-value of the χ^2^ test is shown). *ρ *_*AR(1)*,_ parameter Φ of the autoregressive correlation term AR(1); *ρ *_*MA(1)*_ parameter Θ of the moving average correlation term MA(1). *R*^*2*^_*m*_, marginal r-squared reflecting the proportion of variation explained by fixed effects; *R*^*2*^_*c*_, conditional r-squared indicating the proportion of the variance explained by fixed and random effects. Significance values for the regression estimates: *p < 0.1; **p < 0.05; ***p < 0.01.

^†^ No *p*-value was computed.

**Figure 4:**
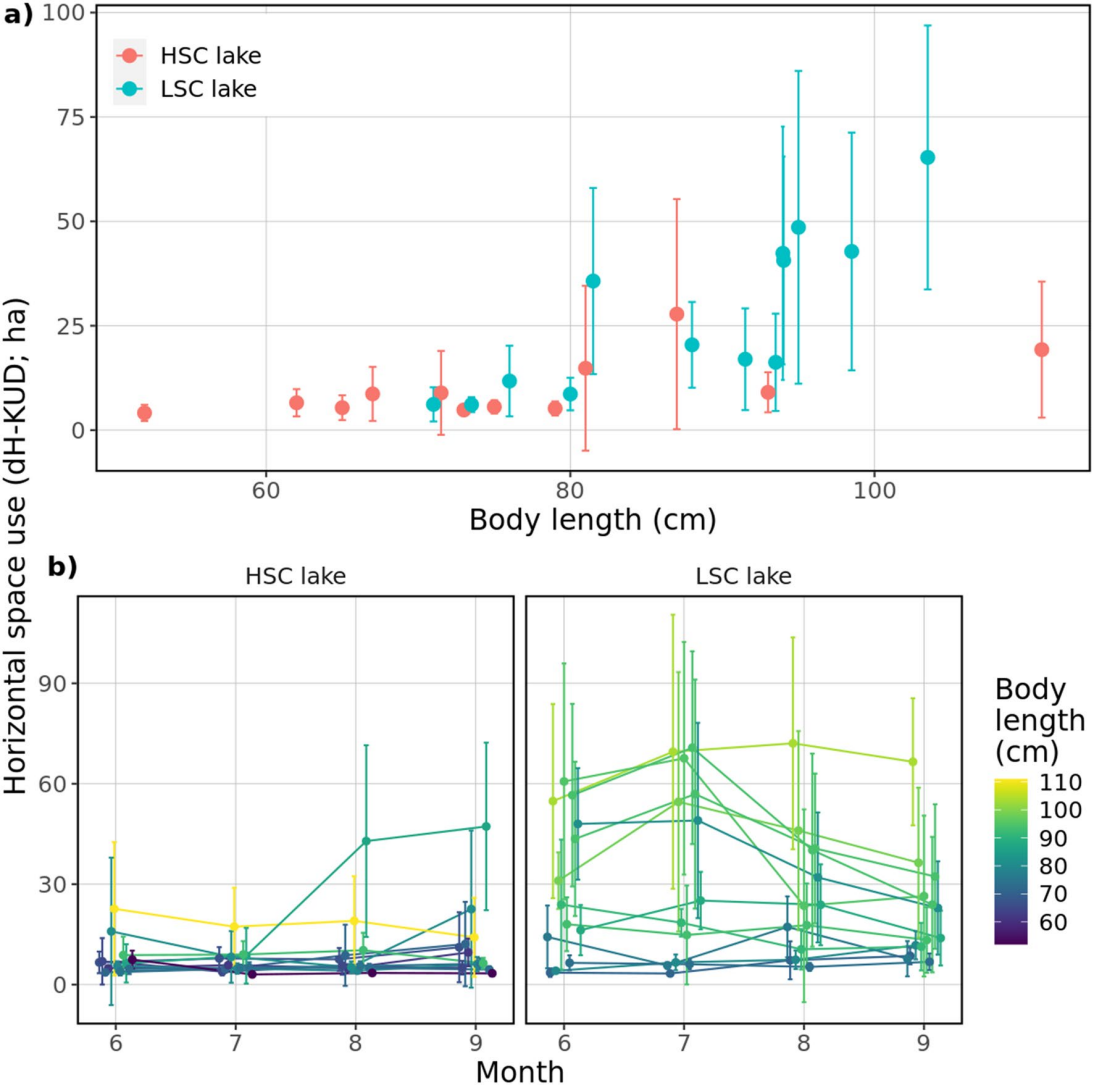
(**a**) Dependence of daily extent of horizontal area (dH-KUD) and body length of observed pike, (**b**) dH-KUD of each individual in each month. Dots represent mean values for the whole observed season/month, error bars denote standard deviation. Colours in (**b**) were set according to body length of tracked individuals.

The extent of explored horizontal area (dH-KUD) was significantly higher in the LSC lake (t = − 3.311, *P* < 0.01), with temporal trends being positive and marginally varying between lakes (time × lake, t = − 1.94, *P* = 0.052, SM1 sec. Extended results). dH-KUD significantly increased with body length in both lakes (t = 3.63, *P* < 0.01) but with a steeper slope in the LSC lake (Least-squares means on body length_slope_ ± SE, HSC lake: 0.14 ± 0.04; LSC lake*:* 0.37 ± 0.05; t = − 3.552, *P* < 0.01) (Fig. 4a). Water temperature was negatively related to horizontal range (t = − 2.42, *P* < 0.05, SM1 sec. Extended results). Repeatability in the LSC lake was more than 1.6 times the amount observed in the HSC lake (*R* ~ 0.49 vs. 0.31) clearly showing more intra-individual variation under low structural complexity (Fig. 4b). These results are consistent with the Spearman rank correlation tests by time periods, for June-July (Spearman’s Rho; LSC lake: ρ = 0.93; HSC lake: ρ = 0.46), June–August (LSC lake: ρ = 0.75; HSC lake: ρ = 0.20), and June–September (LSC lake: ρ = 0.78; HSC lake: ρ = 0.20). The higher coefficients in the LSC lake were associated with greater inter-individual variation in respect to dH-KUD (Fig. 4b).

**Figure 5:**
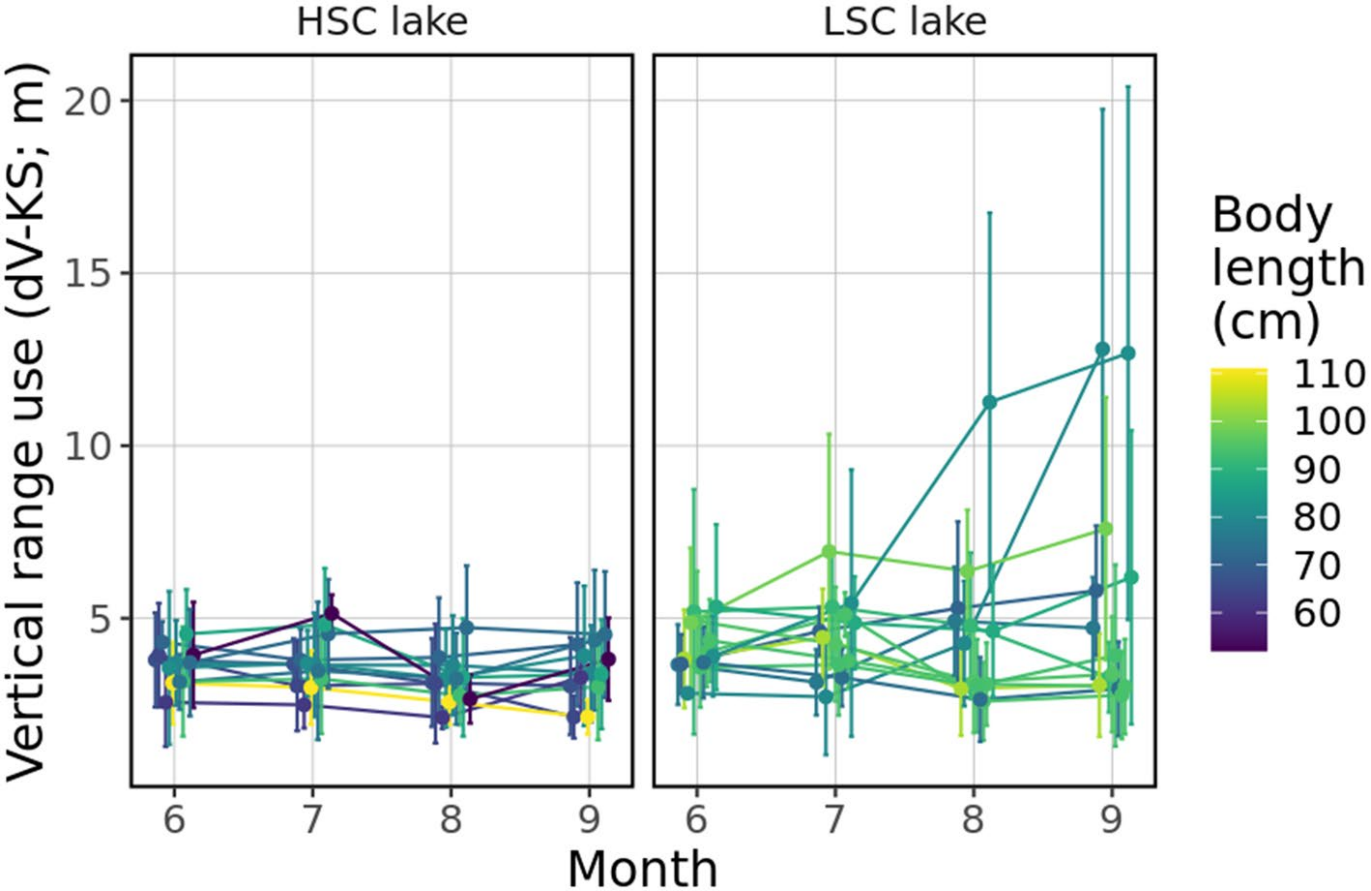
Daily extent of vertical space use for each individual in each month. Dots represent mean values for the whole observed month and error bars denote standard deviation. Colours are set according to body length of tracked individuals.

In general, vertical utilization distribution (dV-KS) was not significantly different between the lakes (t = 1.68, *P* = 0.11), and likewise with temporal trends (time × lake, t = 0.99, *P* = 0.322). It increased as the water temperature decreased irrespective of the lake’s structural complexity (t = − 2.88, *P* = 0.004, SM1 sec. Extended results). Variability in vertical range use over time was marked between lakes, with pike in the HSC lake showing less inter-individual variation than conspecifics in the LSC lake (*R* ~ 0.18 vs. 0.15) (Fig. 5).

**Figure 6:**
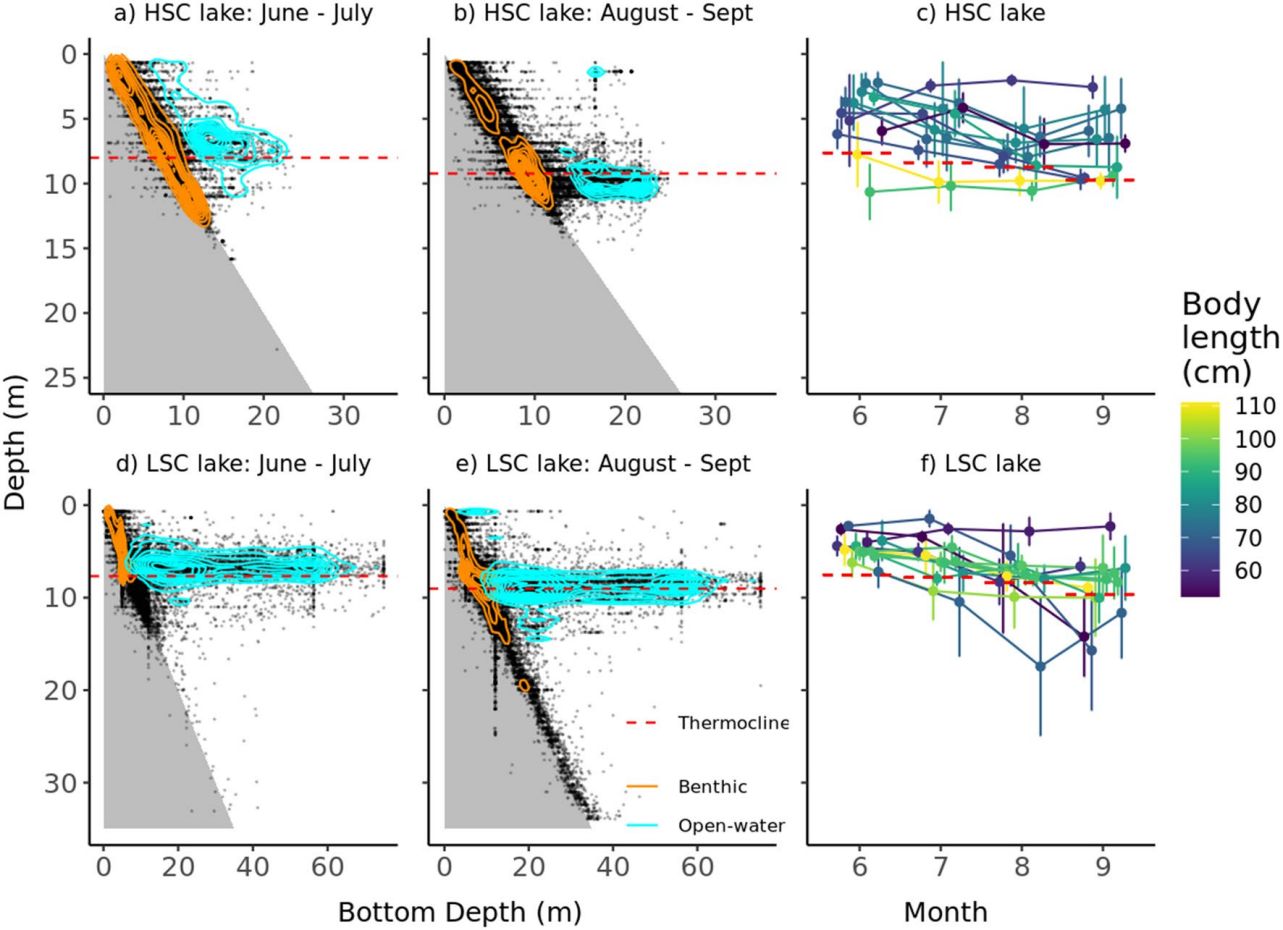
Two-dimensional distribution of all pike positions (dots) in relation to bottom depth for HSC lake (**a**, **b**) and LSC lake (**d**, **e**) and depth use for each individual in each month (**c**, **f**). Isoclines depict the highest concentration of positions in the benthic (orange) and open water (light blue) habitats. Mean thermocline depth within each period is indicated by a dashed line.

Mean daily depth of pike in general increased with time (t = 2.74, *P* = 0.006) but it was not significantly different between lakes (t = − 1.07, *P* = 0.29) and likewise across-lake temporal trends (time × lake, t = − 0.83, *P* = 0.41) (Fig. 6) It showed a positive relationship with body length (t = 2.62, *P* = 0.016) and water temperature (t = 2.15, *P* = 0.031, SM1 sec. Extended results). Inter-individual variation in mean depth across time was very similar in the two lakes (*R* ~ 0.40) (Fig. 6c,f) and differences were mostly due to varying distribution patterns in the water column (Fig. 6a–e). In the benthic habitats, pike were dispersed from the surface down to 12–15 m in the HSC lake throughout the season, while in the LSC lake pike utilized even deeper depths down to 35 m at the end of the summer (likely even deeper but the depth sensor could not record depths below 35 m). In open water, pike were distributed primarily around the thermocline (Fig. 6).

### Activity

**Figure 7:**
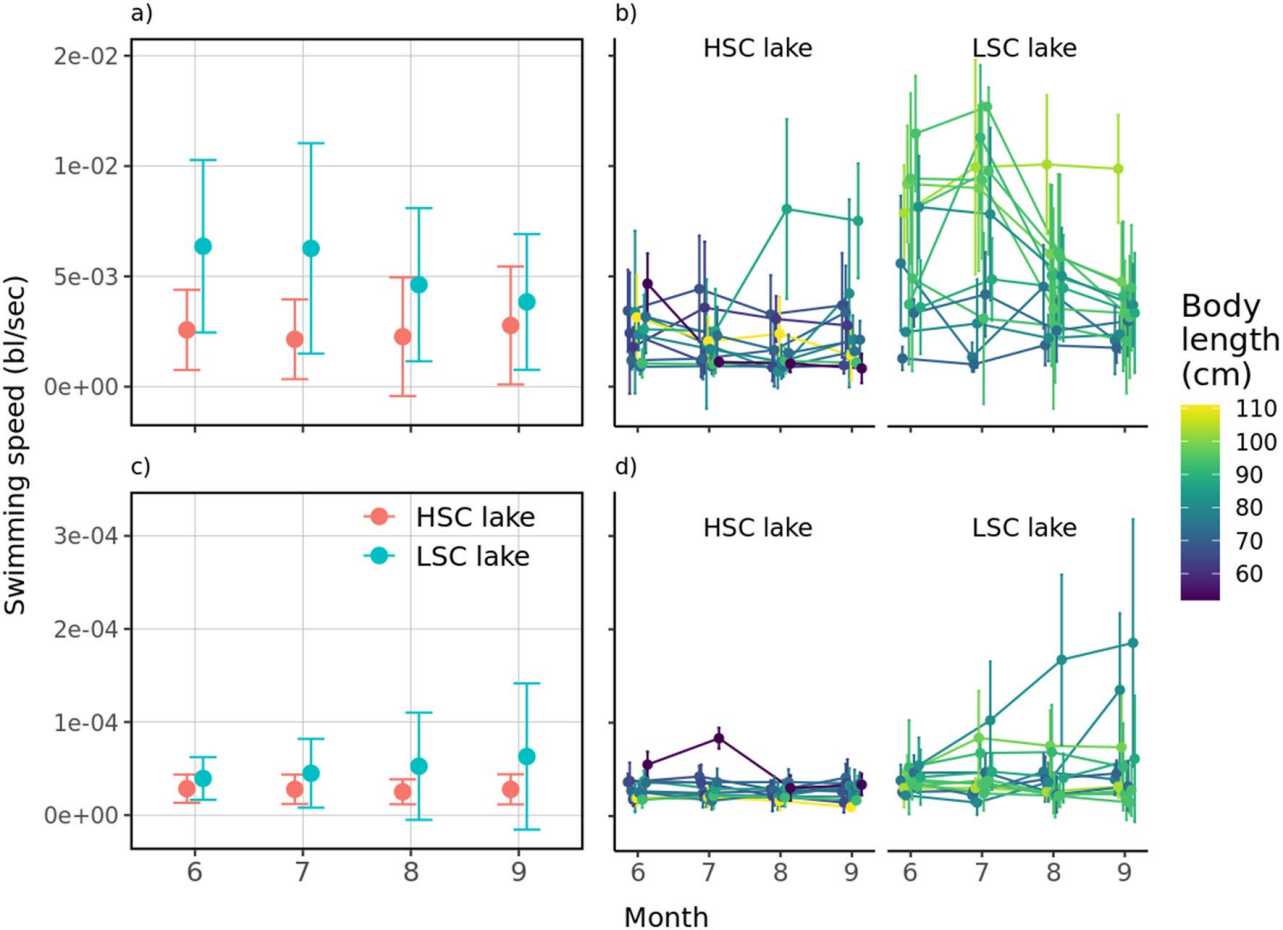
Development of mean horizontal and vertical activity during tracking period. Pooled (**a**) and individual (**b**) horizontal activity; pooled (**c**) and individual (**d**) vertical activity in each month of tracking. Dots represent mean values for the whole observed season/month and error bars indicate standard deviations. Colours in (**b**) and (**d**) were set according to body length of tracked individuals.

Regardless of when it was measured, pike from the LSC lake were generally more horizontally active than those from the HSC lake (t = 4.03, *P* < 0.001) with temporal trends in opposite directions, decreasing in the LSC lake and increasing in the HSC lake (time × lake, t = − 2.48, *P* = 0.013) (Fig. 7a). Irrespective of the lake complexity, body length was positively associated with fish activity (t = 2.34, *P* = 0.028) while water temperature adversely influenced this behaviour parameter (t = − 3.38, *P* < 0.001, SM1 sec. Extended results). Repeatability in swimming activity was generally higher in the LSC lake than in the HSC lake (*R* ~ 0.30 vs. 0.36), consistent with the Spearman rank correlation tests for June-July (LSC lake: ρ = 0.68; HSC lake: ρ = 0.45), June–August (LSC lake: ρ = 0.73; HSC lake: ρ = 0.05), and June–September (LSC lake: ρ = 0.50; HSC lake: ρ = 0.11). These results indeed reveal higher levels of intra-individual variation under low structural complexity in the HSC lake showing significantly less inter-individual variation (Fig. 7b).

In principle, vertical activity was not significantly different between lakes (t = 0.99, *P* = 0.33) nor was their temporal trends (time × lake, t = 1.15, *P* = 0.25). However, we observed that either by ignoring or dropping the time by lake interaction from the model, differences become significant (both: Least-squares means on lake ± SE, HSC lake: 5.2 × 10^–03^ ± 3.2 × 10^–04^; LSC lake*:* 6.1 × 10^–03^ ± 3.1 × 10^–04^; t ~ − 2, *P* < 0.05), suggesting that swimming speeds were on average different in both lakes at the end of the study (Fig. 7c). This also implies that regardless of the measurement time, the LSC lake’s mean vertical activity is always higher than the HSC lake’s. In turn, increased vertical activity was generally associated with decreased water temperature (t = − 3.14, *P* = 0.002, SM1 sec. Extended results) but this effect disappeared after including this variable in an interaction with factor lake making between the lakes variation significant (lake, t = 2.35, *P* = 0.028). Consequently, despite the downward trend in swimming speeds with temperature in the LSC lake, means in this lake kept generally above HSC lake. Yet, even if the temperature by lake interaction was preferred (model 3 vs. model 4, Likelihood-ratio test, χ^2^_11,12_ = 13.47, *P* < 0.001; Table A6, Supplementary material), the temporal effect, while not mutually exclusive, appeared to be a more satisfactory explanation to the observed differences between lakes based on AIC. Inter-individual variation in vertical space use was significantly greater in the LSC lake than in the HSC lake (*R* ~ 7.25 × 10^–05^ vs. 0.51), consistent with the observed patterns of variability over time (Fig. 7d).

### Pelagic habitat use and food resources

**Figure 8:**
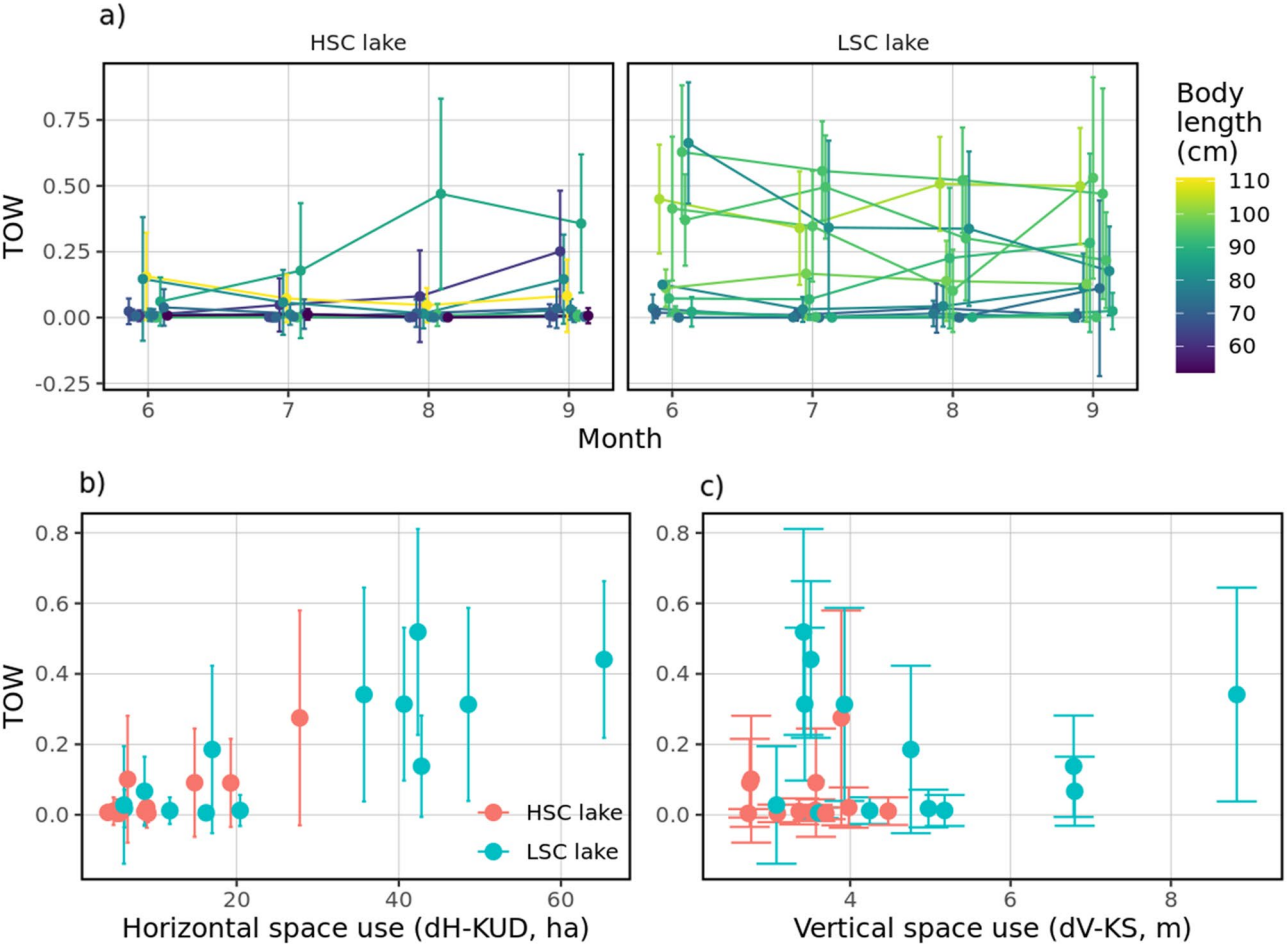
(**a**) Individual development of TOW during tracking period; relation between mean dH-KUD (**b**) or dV-KS (**c**) and mean TOW. Dots represent mean values for the whole observed season/month and error bars indicate standard deviations. Colours in (**a**) were set according to body length of tracked individuals.

The analysis of time spent in open water (TOW) is shown in Table 4 with model selection in Table A7 (Supplementary material). TOW differed between lakes and as a non-linear function of both dH-KUD and dV-KS (Fig. S8, Supplementary material). Overall, TOW (lake_(μ)_, t = 7.99, *P* < 0.001) was higher and more variable (lake_(σ)_, t = 7.70, *P* < 0.001) in the LSC lake and increased over time (time_(σ)_, t = 4.05, *P* < 0.001, Fig. 8a). Higher levels of dH-KUD were correlated with increased likelihood of TOW in both lakes (dH-KUD_(μ)_, t = 7.99, *P* < 0.001, Fig. 8b), while increasing dV-KS was associated with a decreased likelihood (dV-KS_(μ)_, t = − 9.54, *P* < 0.001, Fig. 8c) but increased variability (dV-KS_(σ)_, t = − 5.30, *P* < 0.001), with both effects persisting over time. Only factor lake was significantly associated with each of the ν and τ components representing probability of exclusive use of benthic habitats (p(TOW) = p_0_) or exclusive use of pelagic habitat (p(TOW) = p_1_) respectively. Holding other covariates at fixed values, fish in the LSC lake were less likely to use the benthic habitat only (p(TOW) = p_0_) than fish in the HSC lake (OR 0.721 [0.55–0.95], *P* = 0.02), which was associated with higher dH-KUD (OR 0.21 [0.17, 0.25], *P* < 0.001) and body length (OR 0.841 [0.73, 0.96], *P* = 0.012). Also, fish in the LSC lake were more likely to use the pelagic habitat only (p(TOW) = p_1_) than fish in the HSC lake (OR 16.35[1.83, 146.33], *P* = 0.0125) irrespective of dH-KUD and body length.

**Table 4:** Final model analysing pike use of pelagic habitat in both lakes.

Dependent variable: TOW													
Parameter	Variable	b	SE	t	*p*	*edf*	σ^b^	ϓ	σ^2^	τ_00_ _tag_id_	τ_11_ _tag_id.time_	ρ_01_ _tag_id_	*R*
*μ* (logit)	(Intercept)	− 1.63	0.06	− 26.1	** < 0.001**								
	pb(time)	0.003	0.001	4.05	** < 0.001**	10.48	0.16	15.14					
	Lake	0.76	0.09	7.99	** < 0.001**								
	pb(dH-KUD)	0.74	0.03	27.10	** < 0.001**	6.19	0.05	180.92					
	pb(dV-KS)	− 0.22	0.02	− 9.54	** < 0.001**	15.42	0.55	1.34					
	pb(time) × lake	− 0.01	0.001	− 4.74	** < 0.001**								
							Random effects		0.41	0.84	6.52 × 10^–05^	− 0.53	0.67
*σ* (logit)	(Intercept)	− 2.04	0.09	− 22.1	** < 0.001**								
	pb(time)	0.01	0.001	6.30	** < 0.001**	14.09	0.47	1.75					
	Lake	0.99	0.13	7.70	** < 0.001**								
	dH-KUD	0.02	0.03	0.76	0.447								
	pb(dV-KS)	− 0.15	0.03	− 5.26	** < 0.001**	12.89	0.37	2.92					
	pb(time) × lake	− 0.003	0.002	− 2.25	**0.025**								
							Random effects		0.40	0.63	2.85 × 10^–05^	− 0.59	0.61
ν (log)	(Intercept)	0.06	0.15	0.37	0.71								
	pb(time)	0.01	0.002	3.63	** < 0.001**	6.06	0.15	35.99					
	Lake	− 0.33	0.14	− 2.33	**0.02**								
	pb(dH-KUD)	− 1.58	0.102	− 15.50	** < 0.001**	4.05	0.08	113.75					
	Body length	− 0.17	0.07	− 2.51	**0.012**								
							Random effects		0.84	1.81	1.34 × 10^–05^	− 0.35	0.68
τ (log)	(Intercept)	− 9.26	1.73	− 5.35	** < 0.001**								
	pb(time)	0.03	0.02	1.81	0.071	11.29	68.4	1.67 × 10^–05^					
	Lake	2.79	1.12	2.50	**0.013**								
							Random effects		0.08	9.62			0.99
	GAIC	172.5											
	GDEV	− 271.1											
	SBC	1408.9											
	pseudo-R^2^	0.71											

Relationship of behavioral traits (dH-KUD, dV-KS) and body length on pike use of pelagic habitat as a function of the time spent in open water (TOW)**.** The model fits a zero–one beta inflated distribution *BEINF*(μ, σ, ν, τ) defined by separate linear predictors or (penalized P-splines) smooth terms (*pb*) for all four parameters, and an additive random effect term with autocorrelation, with either a random slope-intercept (μ, σ and ν) or a random-intercept only (τ). *b*, variable estimate (z-score); *SE*, standard error; *t*, t-statistics; *p*, *p*-value (in bold, *p*-values lower than 0.05); *edf,* effective degrees of freedom of the smooth terms*; σ*^*b*^, Random effect parameter of the smooth term; *ϓ*, smoothing parameter. Random effects were calculated as in Table 2; *GAIC*, Generalized Akaike information criterion; *GDEV,* global fitted deviance; *SBC*, Schwartz Bayesian Criterion; *pseudo-R2,* generalized pseudo R-squared.

**Figure 9:**
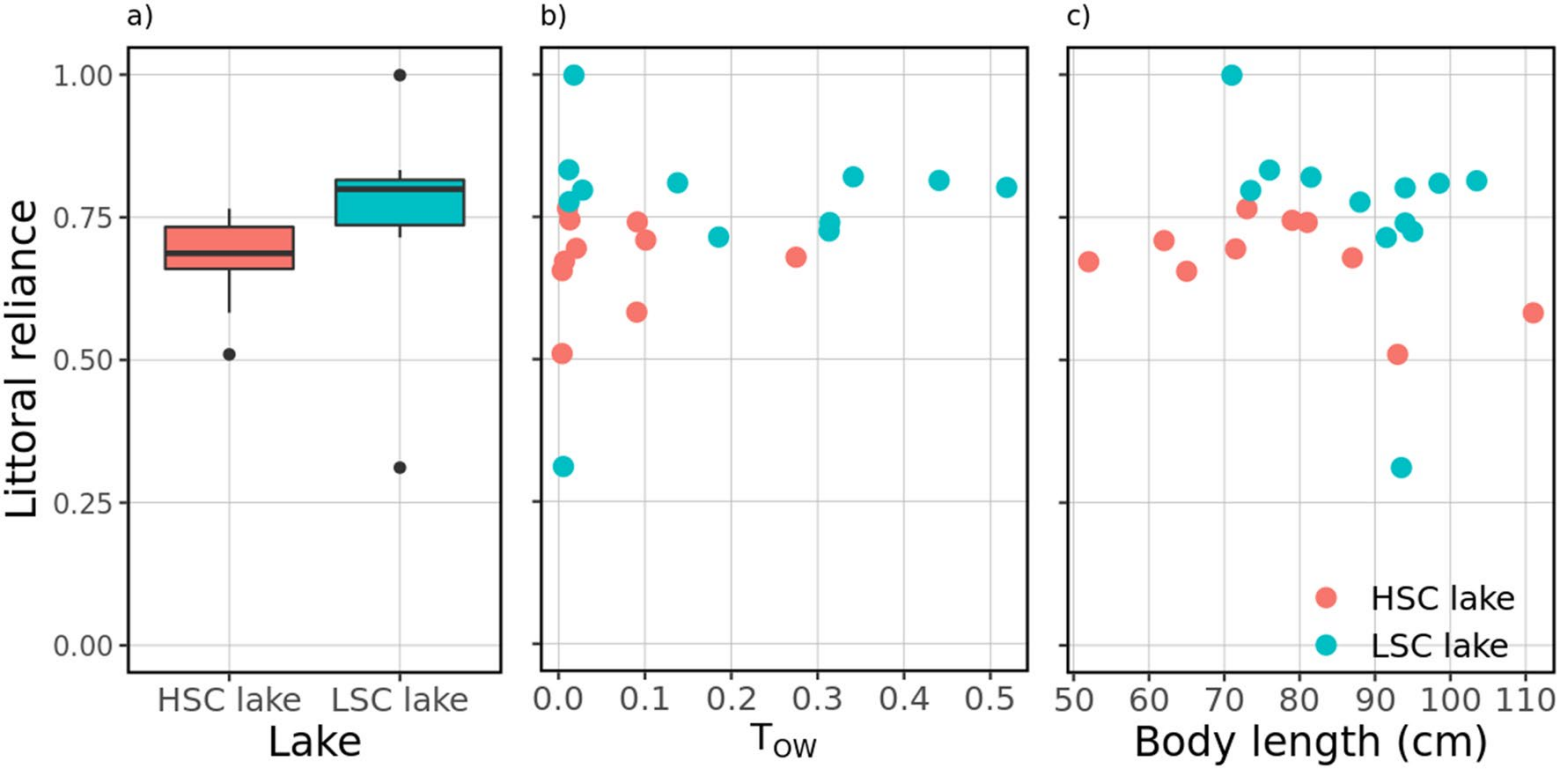
Pike littoral reliance as a function of structural habitat complexity (Lake as a factor) (**a**), open-water use (based on telemetry data, (**b**) and total length (**c**) in HSC and LSC lakes. The individual with the lowest littoral reliance estimate was excluded from the modelling due to its high influence on final results.

A significant effect of lake and a negative effect of body length were found on pike littoral reliance (Fig. 9, Tables 5, 6). The second best equally supported model (ΔAIC = 1.42) also suggested a positive effect of open water use on littoral reliance (Table 5). In both lakes, pike seemed to shift to a less littoral (i.e., more pelagic) diet with increasing body length (Fig. 9). Unexpectedly, pike tended to rely more on littoral resources in the LSC lake (Fig. 9), where the individual with the lowest littoral reliance estimate (excluded from modelling) also showed the lowest use of open-water areas. In fact, the stable isotope data indicates that only this single pike relied more on pelagic than on littoral food resources (further results of SIA analysis are given in SM1, sec. Extended results).

**Table 5:** Summary of the best model predicting pike trophic niche.

Parameter	Estimate	SE	*P*	− 95% CI	+ 95% CI
Intercept	0.658	0.022	< 0.001	0.611	0.705
Lake	0.157	0.032	< 0.002	0.091	0.224
Length	− 0.083	0.032	0.018	− 0.151	− 0.016

Littoral reliance LR calculated with a two-source isotopic mixing model^48^ as a function of structural habitat complexity (Lake as a binary factor) and pike body length (in mm). Continuous variables are standardized to allow direct comparison of effect sizes. Standard error (SE), *P*-values and 95% confidence intervals (CI) for each parameter are shown.

**Table 6:** Model selection tables for 95% confidence sets of top candidate models predicting pike littoral reliance (LR).

Intercept	Lake	OWU	Length	K	AIC	ΔAIC	Wi
0.66	+		− 0.08	4	− 48.70	0.00	0.67
0.66	+	0.03	− 0.10	5	− 47.30	1.42	0.33

LR was tested against structural habitat complexity (Lake as a binary factor) as well as pike open-water use (OWU, based on telemetry data) and body length (in mm). Parameter estimates for model terms included in the models, number of parameters (K), AIC, AIC difference from best model (ΔAIC), and Akaike weights (weights) are shown. Continuous variables are standardized to allow direct comparison of effect sizes.

### Growth

The final and alternative models of pike growth are shown in Table A8 (Supplementary material). Growth varied between lakes (Least-squares means ± SE, HSC lake: 90 ± 16.5; LSC lake: 142 ± 13.5; t = − 2.157, *P* = 0.045), and with the age of individuals (t = − 3.90, *P* < 0.01), while no significant correlation was found with body length. Given a significant crossover interaction between dH-KUD and dV-KS (dH-KUD × dV-KS, t = 2.448, *P* = 0.025), we ran an interaction analysis to further determine the nature of the relationship between those variables and growth rate. The result showed a conditional effect of dH-KUD on growth as a function of dV-KS with growth increasing as dH-KUD increases at higher dV-KS values and decreasing with dH-KUD at lower dV-KS values (see SM1 for further details, sec. Analysis of growth rate using linear regression).

## Discussion

Structural complexity was found to have a strong directional influence on multiple pike behavioural traits, with clear differences between the LSC and the HSC lakes. As hypothesized, pike exhibited higher horizontal space use and higher activity in the LSC lake as compared to the HSC lake. Moreover, the increased space use with increased activity also indicates that higher activity levels were associated with exploring new areas rather than revisiting already visited areas. Exploration of more extended areas was positively related to pike size and linked with higher use of open water, also reflected in lower littoral reliance in the diet. There was a high degree of consistency in individual behaviour, individuals having high space use, activity and/or open water use in one month, also had so in the next month (high repeatability) when there was a high degree of between-individual variation. Inter-individual differences varied between lakes, with activity and exploration of horizontal space showing higher individual rank correlation between months in the LSC lake where the between-individual variation was high. With low-between-individual variability, rank correlation between months also tended to be low. Although rank correlation decreased over time in both lakes, the correlation was consistently much higher in the LSC lake. Contrary to what was expected, individual growth was overall higher in the LSC lake, indicating that pike in the LSC lake were able to more than compensate for increased activity costs by increased foraging success. Despite observed differences in pike behaviour and growth, stable isotopes showed a low degree of specialization and a high dependence on littoral food sources in both lakes.

### Horizontal space use and activity

We found that activity and horizontal space use and activity were higher with lower structural complexity. Predator space use is largely driven by prey abundance and predator body size^71–73^, and a positive relationship between pike body size and horizontal space use has been previously documented in numerous studies^23,31,74–77^. Previous research on predators capable of performing both active pursuit and ambush strategies indicated that a switch between these modes was primarily linked to prey density or prey type^78–81^. However, contrary to general expectations, prey abundance alone cannot explain the larger space use and active foraging of pike in the LSC lake. Comparison of prey density and type in pike-preferred littoral habitat showed large similarities between lakes (we detected even higher prey abundance in the LSC lake in 2015). Moreover, similar alteration of activity, forage mode or space use with habitat complexity has been observed in several fish species^7,82–84^. As in our study, Ahrenstorff et al.^82^ found that the home range of largemouth bass (*Micropterus salmoides*) increased and that the bass switched from ambush predation to active searching behaviour when structural complexity decreased. Pike body morphology is clearly adapted to ambush and attack foraging, which is also regarded as the preferred foraging strategy of pike^85^. But structurally complex macrophytes also serve as cover/camouflage for ambush pike as well as attraction for prey fish, and in the lack of macrophytes pike cannot rely on camouflage, as they will be significantly more easily spotted and avoided. This may force pike into a more active search to find local distributions of prey. The higher availability of pelagic prey may increase the potential profitability of pelagic foraging, but this is not a necessary condition to explain altered behaviour from ambush predation to active search. Therefore, we propose differences in habitat complexity between lakes as a crucial driver for observed behavioural differences among pike. Ambush individuals (prevalent behaviour in the HSC lake) have camouflage, use a small area of the macrophyte bed and limited vertical movement as they wait to ambush their prey. On the contrary, pike in the LSC lake cannot rely on camouflage as they will be significantly more easily spotted and avoided in the lack of macrophytes, and are forced into a more active search to find local distributions of prey. The pattern of inter-individual differences and repeatability also suggest that while in their preferred macrophyte habitat, pike behaviour has low variability. In contrast, when pike are forced away from the ambush-strategy, inter-individual differences increase, indicating a wider range of hunting strategies with individual specialization into a pattern repeated over time.

Intraspecific competition is considered as an important driver of individual behavioural and can induce inter-individual variation^15,86^. Considering similarity in prey availability, intraspecific competition for prey was more likely to be higher in the HSC lake (larger pike stock, see Study site description), where the ratio of prey per pike capita was lower. Besides prey resources, pike in the LSC lake may compete more for available preferred structured habitats than in the HSC lake^18^. If this were true, we would expect a high variation in horizontal space use depending on whether an individual will find a structured spot with/without a conspecific. However, this expectation is not supported by the observed high repeatability of horizontal range use in the LSC lake. High repeatability demonstrates stable individual patterns, with individuals preferring either an active or a passive hunting strategy. Besides that, large individuals with higher competitive abilities adopted an active hunting mode, whereas smaller individuals retained low home ranges and activity levels in both lakes. Therefore, we argue that switching to an active hunt mode to find more prey when structures for camouflage are not available is a more probable explanation than intraspecific competition forcing individuals to less profitable environments.

### Vertical space use and activity

Our results showed that, despite similarity in vertical space use and depth preference in both lakes, vertical activity was higher and several individuals performed very deep dives in the LSC lake. Temperature conditions within the water column were similar in both study lakes, and pike mostly preferred the area from the surface down to the upper hypolimnion in benthic habitats and around the thermocline in open water. Recent behavioural studies have highlighted that exploration of movement in the vertical dimension is important for understanding animal habitat use^87^. The limited available information suggests that water temperature is an important driver of pike vertical distribution^47^, with pike normally preferring relatively cold parts of the water column^36^. This would force pike deeper as the surface waters get very warm, as we indeed observed in our study. Bioenergetic advantages of heat gain from “sun basking” in surface waters has been suggested in some circumstances^47^, however, this may occur only at lower water temperatures than observed in surface waters in our study in July and August. Very warm surface waters may also explain why the development of higher structural complexity at depths shallower than 5 m in the LSC lake towards the end of the season was not accompanied by increased pike association. Higher vertical activity in the LSC lake meant individuals were more active within the vertical space, which is in concordance with higher activity in horizontal space and reflects the active hunting strategy in both spatial dimensions.

### Open water and prey distribution

We propose macrophytes to act as a crucial driver of pike activity and space use in both lakes, without differences in prey distribution being a prerequisite. But prey distribution may clearly play an important role for pike habitat use^88^, since there would be no benefit of pelagic prey search without pelagic prey present. In the LSC lake, use of extensive horizontal areas was tightly linked with frequent use of open water, contrary to the HSC lake where open water areas were rarely explored. This corresponded with higher abundance of pelagic prey fish in the LSC lake as compared to the HSC lake. When foraging in the pelagic areas, there is no structural complexity to aid cover or camouflage for ambush predation, and a search-based foraging mode is likely to be more effective. Low environmental complexity may induce individual differentiation in food source and a higher inter-individual trophic niche^18,33,35^, which are in line with our observations in this study.

### Potential dietary specialization

Our results showed higher inter-individual differences in horizontal space use among pike in the LSC lake, suggesting a link between behaviour and prey specialization. However, our results showed minor, if any, individual dietary specialization. Even the pike individual with the highest open water use depended strongly on littoral food resources, and only one individual had higher pelagic than littoral reliance. These findings suggests that, even though pike in the LSC lake had a wider spatial niche, the littoral prey fishes like tench, perch, rudd, roach and ruffe were still the most important prey^17,34,35^. The results from telemetry and stable isotope analyses are not directly comparable, since the stable isotopes reflected the time before the telemetry study. However, we do not expect substantial changes in pike diet and behaviour because the environmental conditions in both study lakes had been similar the year before this study. Vejřík et al.^35^ studied the diet and trophic position of pike in our study lakes, and also found that pike in the LSC lake had a wider trophic niche as well as a lower trophic position than pike in the HSC lake. Together with our findings, this indicates an existence of open water and littoral prey specialists in pike in the LSC lake, and also that alteration of foraging mode (from ambush behaviour to search behaviour) is not necessarily accompanied by a switch from littoral to pelagic prey reliance. Rather, the structural complexity in the littoral zone seems to be the main key for altered behaviour in pike.

### Pike activity, energy gains and growth

The growth rate of pike was higher in the LSC lake, contrary to our expectations based on higher metabolic costs associated with higher activity. Moreover, growth was positively correlated with increased activity and space use in both horizontal and vertical dimensions. Such findings suggest that increased activity was more than compensated for by increased energy intake. As with the stable isotopes, our growth analyses reflected the time before the telemetry analyses. But as the effects of individual and size on behaviour were consistent, we assume there was a good correspondence between growth in the previous year and activity in the present year. The switch between ambush and active mode does not imply a large increase in expended energy in ectotherms^80,81^, and Lucas et al.^89^ calculated that the cost of activity comprised only up to 15% of standard metabolism for even relatively active pike. Given that the prey density was similar in both study lakes, the higher pike activity in the LSC lake would imply an increased prey encounter rate^90^, potentially leading to higher ingestion rate if capture rate was not severely reduced. Recent studies have shown contrasting evidence for the relationship between pike growth and activity. Laskowski et al.^21^ found no correlation between pike behaviour and growth rate in a standardized assay. In contrast, Nyqvist et al.^30,91^ found higher activity to support an increased growth rate of riverine pike juveniles, whereas Kobler et al.^31^ found active and opportunistic adult pike to grow faster than the less explorative individuals in a small lake. Savino and Stein^9^ found that pike had higher attack rates and hunt success in a homogeneous environment. Our results support the latter findings, strongly suggesting altered behaviour as the mechanism underlying the observed higher growth in the LSC lake even under similar prey availability.

### Contribution of pike origin

We were not able to distinguish between autochthonous (born in lakes) and allochthones (stocked) pike, and some large pike tagged in the LSC lake may have had a stocking origin. Recent research showed that translocation to a novel environment might influence space use for up to several months^23^. Pike stocking ceased 2 and 10 years before this study in the LSC and HSC lakes, respectively, therefore there would be a large proportion of lake-born recruited pike in both lakes, and translocated pike would have had years to adapt their behavior to local conditions. Moreover, pike in our recently created study lakes had a similar origin and thus no time for genetic adaptation to the new local conditions. Since the allometric influence on activity and space use were similar in both lakes, we do not believe that potentially stocked fish had any influence on our results and conclusions.

### Caveats

We argue that the substantial differences in the level of structural complexity between our two study lakes is the most plausible explanation for between-lake differences in pike behavior. However, our study was an uncontrolled experiment with no repetition (due to obvious limitations in availability of lakes with such unique similarity/dissimilarity conditions and large fish tracking demands). Therefore, we could not mechanistically demonstrate that the level of structural complexity induced behavioural differences among pike. In addition, our study lakes differed to some extent by other environmental parameters (e.g., water transparency, maximum depth, prey species composition and oxygen availability) and there may be even other factors contributing to between-lake differences in pike behaviour. Thus, further research should be conducted in more controlled environments to obtain more precise mechanistic explanations for the detected behavioural differences.

## General conclusions

Our study showed that structural complexity can have large impacts on behaviour of apex predators in natural conditions. The observed behavioural differences of the apex predator may have contrasting, potentially cascading impacts on lower trophic levels. Different predator behaviour may favour different prey behaviour, or species and per capita consumptive effects of pike on lower trophic levels must differ in our study lakes. Our results suggest that higher activity levels and thus energy expenditure can be associated with higher growth, which must be balanced by increased consumption. On the other hand, piscivorous fish in habitats with low structural complexity may be more vulnerable to fishing mortality, since the increased activity implies increased encounter probabilities with anglers and/or fishing nets^92^. Ecosystem effects of observed behavioural differences are beyond the scope of this paper and further research is strongly required in this respect, but we argue that the design of concurrently comparing lakes with contrasting structural complexity has a large potential for such research.

## Supplementary Information

Supplementary Information 1.

Supplementary Information 2.

## Data Availability

The datasets used and analysed during the current study are available from the corresponding author on reasonable request.
