# Supplementary material for: Contrasting structural complexity differentiate hunting strategy in an ambush apex predator
Source: Sci Rep. 2021 Sep 1;11:17472. doi: 10.1038/s41598-021-96908-1 (PMC8410764; doi:10.1038/s41598-021-96908-1)
Supplement: Supplementary file 1 — Supplementary Information 1. [file 41598_2021_96908_MOESM1_ESM.docx]

## Supplementary Material

#### APPENDIX: Material and methods

Data processing

Filtering of positions estimated by the U-MAP software

U-MAP (User Managed Acoustic Positioning) is a proprietary software from Lotek Inc. that processes transmitter detection data collected with autonomous Lotek WHS-receivers. The detection data recorded by each WHS receiver include tag ID, time of arrival (TOA) and relative signal strength or power of the detected signal. U-MAP uses these data to estimate transmitter positions by means of multilateration on TOA data for transmissions that were detected at three or more receivers. The algorithms solve non-linear equations involving hydrophone positions and the difference in time of signal arrival (TDOA) at each hydrophone detecting the transmission. Depending on the number and geometry of the hydrophones involved in the position calculation, the equation may have multiple solutions ^1^. These are referred to as twin solutions (or shadow solutions). U-MAP saves all twin solutions to the output file, and it is up to the user to choose, or filter, the correct position between the twin positions. Moreover, the use of TDOA implies hyperbolic equations, which also tend to give large errors for certain receiver and transmitter configurations. These configurations depend on the receiver array and the transmitter location, but also on stochastic variation in which receivers that detects the signal. Two successive position estimates may therefore differ in location although the transmitter did not move, if the receivers involved in the position estimate differ. Position estimates produced by programs using TDOA-methods, such as U-MAP, therefore requires filtering by post-processing in order to eliminate erroneous position estimates.

The performance of the position estimates and potential filters were evaluated by short terms tag-tows from boat with high-precision GPS-device above the towed transmitters, by several stationary reference tags throughout the study (Fig. S1), and by visual inspections of all fish tracks and filter results (Fig. S2).

The filtering procedure required several steps to ensure the best error exclusion:

1. Calculation of detection rate D_r_ and detection mean power D_p_ at each receiver by 20 minute intervals for the transmitter in focus. Detection rate and detection power are expected to be highest closest to the receiver ^2^. These two measures were then combined into one receiver signal scale Ds by scaling each of them before adding them together;
   $D_{s}=scale\left( D_{r} \right)+scale(D_{p})$. Scaling here means centring and dividing by the standard deviation.
2. Calculation of distance weights D_w_ for later use in calculation of regression weights, by the use of the logistic function: $D_{w,i}\frac{1}{{1+2\cdot0.001}^{\frac{(D}{1500}-0.6)}}$, where Di is distance from the receiver i to the position estimate. Parameters where subjectively fitted to obtain a desired logistic shape with high weights to receivers closer than 500 m to the position estimate, and low weight to receivers farther than 1000 m away from the position estimate.
3. Calculation of regression weights R_w_ were then done for each U-MAP position estimate, by first ranking all receivers from highest to lowest Ds value within the current 20 minutes period. The Ds for the highest ranked receiver was then used together with the distance weight Dw for this receiver to obtain _Rw_ through the function $R_{w}=D_{w}(1+D_{s})$
4. Lake shoreline exclusion: All position estimates farther than 50 m outside the lake shoreline polygon were excluded from further filtering, and marked as false position estimate.
5. Calculation of gam-models (generalized additive model^3^) and gam-predictions for east and north-directions. This was done for successive periods of six hours, with one extra hour of data before and after the end of each period included in the gam-regression to stabilize the ends. The resulting gam-models was then used to predict position (Eastgam, Northgam) within the six-hour period at the same time points as the U-MAP estimated positions. Gam residual was calculated as the distance between the U-MAP position estimate and the gam prediction, including residuals for positions excluded from the gam regression. The gam-formulation was y ~ s(time), where y was either east or north, and s was the smooth term specified with a cubic spline regression model. K, the dimension of the basis used to represent the smooth term, was set as a function of the n observations included in the regression period, with K as the nearest integer to n/15, but with a minimum of value of 3.
6. For U-MAP twin positions (i.e. positions with equal timestamp), the position with the lowest gam-residual was flagged as a true position, the other as false.
7. In order to remove heavy outliers with a strong influence on the gam-regression, U-MAP positions with gam-residuals > 1000 m were flagged as false.
8. A second gam-regression was now performed, repeating point 5 but excluding all positions now flagged as false. In addition, positions with gam-residuals > 150 m were excluded from the input data to the second gam regression. New gam-predictions were made for all time points of U-MAP position estimates.
9. For U-MAP twin positions (i.e. positions with equal timestamp), the position with the lowest gam-residual was flagged as a true position, the other as false.
10. Positions with gam-residual larger than 100 m were flagged as false. U-Map position estimates, gam predictions and gam residuals were stored together in the database, such that the gam residual threshold for position acceptance could later be modified if desirable.

The number of positions varied over time, as well as the relative amount of poor position estimates. This is easy to handle for a tag with known position (Fig. S1a-c), but when one does not know the true position one has to rely on filtering methods using information from the detection data. The DOP measure provided by U-MAP had poor association with the position quality, as position estimates with low DOP-value could be far from the true position, and positions estimates with high DOP-value could be close to true position (Fig. S1d). This filtering procedure described above did a great job in sorting out good from bad position estimates, and in general kept > 85 % of the positions flagged as true positions.

#### Statistical analyses

Extended Methods

Identifying interactions with Random Forest

RF is a machine learning algorithm consisting of a collection of classification trees that grow from the root node by means of bootstrap sampling of the observed data, using a subset of predictors at random to define the best cut on each node (see e.g.^4,5^ for reviews on theoretical and empirical aspects of RF, respectively). An advantage of this method is that it can detect variables with individual weak effects but significant when interacting with other variables and thus it is suitable to assess meaningful interactions between predictors ^6^. For the analysis of our dependent variables the variable importance measures (VIMs) used to assess predictors’ pairs were both the conditional minimal depth and the number of occurrences of interactions across trees. Interactions were ranked from higher to lower likelihood of occurrence and in decreasing mean conditional minimal depth. We split the data into training and test sets representing 60% and 40% of the total dataset. The RF analysis was ran on the training set with a total of 5,000 trees (ntree = 5000) for each forest and the default "top_trees" option to penalize interactions with less frequency of occurrence than the best represented interaction. Each tree was assessed with an Out-Of-Bag (OOB) sample of the test set.

Model selection and inference

To determine the overall support to our hypotheses, we performed selection and inference of linear models and linear mixed-effects models based on different combinations of predictors. In model inference, each independent variable is assigned an importance estimate; variables appearing in largely weighed models are conferred relatively high importance and strong support. Following model selection based on AIC all models were compared using log-likelihood ratio tests (LRTs) to test the interaction effects from the RF analysis. Given two equally likely models (within ∆AICi ≤ 2) the IC criterion prevailed but further comparisons between each two nested models (with one or more parameters in common) allowed us to test alternative hypothesis about the inclusion of interaction terms. This allowed us to discriminate between models to comprise more variability in the candidate sets. We then built 95% confidence sets based on the sum of probabilities associated with the presence or absence of predictors totalling 0.95 (i.e. cumulative AICc weight, ∑wi ≤ 0.95). LRTs were performed using the lrtest() function in R package lmtest 7. In the presence of an interaction term in the best-fit model, we ran post-hoc tests and further trends analysis using the emtrends() and emmip() functions in the emmeans package8.

GAMLSS model of pelagic habitat use (TOW)

Model definition

TOW is a proportional variable, continuous in the interval (0, 1) and with additional probabilities at zero and one, which could depend on the continuous predictors dH-KUD, dV-KS, body size, and an additional interaction term (time × Lake). We fitted GAMLSS models ^9^ assuming a mixed continuous-discrete beta inflated distribution of the response variable, denoted by TOW ~ BEINF(μ, σ, ν, τ) for 0 ≤ TOW ≤ 1, with four parameters, two location and scale parameters (μ , σ), and two shape parameters (ν, τ). The distribution parameters were modeled with the default link functions of the family BEINF: two logit links for the continuous values (0 < TOW < 1) of the beta distributed variable BE(µ, σ) (Eq. 10.46 in ^10^, pp.215), with mean E(TOW) = μ and variance/standard deviation Var(TOW) = σμ(1 – μ), i.e., sub-models ‘mu’ and ‘sigma’ in R; and two log links for the shape parameters (ν, τ) related to the skewness and kurtosis of the distribution, which model the probabilities at the endpoints of the interval (p0, p1), i.e., sub-models ‘nu’ and ‘tau’ in R. Therefore, the fitted GAMLSS model consists of four sub-models, one for each parameter of the distribution, with two logit models for the mean and variance on (0, 1) and two log models for the probabilities at 0 and 1, which can be expanded in the following equations (see ^9^):

$g_{1}\left( \mu\right)=\eta_{1}=X_{1}\beta_{1}+\sum_{j=1}^{J_{1}} h_{j1}\left( x_{j1} \right)$ (1.1)

$g_{2}\left( \sigma\right)=\eta_{2}=X_{2}\beta_{2}+\sum_{j=1}^{J_{2}} h_{j2}\left( x_{j2} \right)$ (1.2)

$g_{3}\left( \nu\right)=\eta_{3}=X_{3}\beta_{3}+\sum_{j=1}^{J_{3}} h_{j3}\left( x_{j3} \right)$ (1.3)

$g_{4}\left( \tau\right)=\eta_{4}=X_{4}\beta_{4}+\sum_{j=1}^{J_{4}} h_{j4}\left( x_{j4} \right)$ (1.4)

where μ, σ, ν, τ are the distribution parameters vectors of length$\eta_{k}$, with link function gk, X_k_ are fixed parameter estimate design matrices incorporating the linear predictor terms that can affect the distribution parameters (e.g., factor Lake); $\beta_{k}$ are parameter vectors of the linear (z-score) coefficients, $h_{\mathrm{jk}}$ are additive smoothing functions (penalized P-splines) for fixed explanatory variables $X_{\mathrm{jk}}$ evaluated at vector $x_{\mathrm{jk}}$ of length $\eta_{k}$, which are a function of the random effects such as $h_{\mathrm{jk}}\left( x_{\mathrm{jk}} \right)=h_{\mathrm{jk}}=ϓ_{jk}$, where $ϓ_{jk}$represents the random additive term including the random (slope-intercept) effects and an ARMA autocorrelation structure of order (p=0, q=1).

According to (^10^ Equation 10.47, pp.215), the probability density function of the beta inflated distribution for the response variable TOW is given by the following expression:

$f_{Y}\left( TOW|\mu,\sigma,\nu,\tau\right)=\left\{ \begin{aligned} p_{0} \\ \left( 1-p_{0}-p_{1}) \right. \\ p_{1} \end{aligned} \right.f_{W}\left( \mathrm{TOW} \right) \begin{matrix} if TOW=0 \\ if 0<TOW<1 \\ if TOW=1 \end{matrix}$ (2)

for 0 ≤ y ≤ 1, where both $f_{W}\left( TOW \right)\sim BE\left( \mu,\sigma\right)$with 0 < μ < 1, 0 < σ < 1, and the inflated beta with ν > 0, τ > 0 shape BEINF(μ, σ, ν, τ) with parameters μ, σ, ν = p0/p2, τ = p1/p2 and expected mean E(TOW) = (τ + μ)/(1 + ν + τ), where p2 = 1 – p0 – p1, and p0, p1 are non-zero probabilities at zero and one relative to p(0 < TOW < 1) such as:

$p\left( TOW=0 \right)= \frac{\nu}{\left( 1+\nu+\tau\right)} for 0 < p_{0} < 1$ (3)

$p\left( TOW=1 \right)= \frac{\tau}{\left( 1+\nu+\tau\right)} for 0 < p_{1} < 1-p_{0}$ (4)

Fitting a base model with autocorrelation

Within the GAMLSS framework the random effects are estimated by maximizing a penalized likelihood function ℓp (Eq. 6 in ^9^), which implies that dropping a continuous predictor from the model can modify the penalization of the random effect and discard its inclusion even being significant^9^. Thus, rather than backward removal of terms, to find the best GAMLSS model we evaluated and compared different models following a stepwise selection procedure. We started from a null model (i.e., intercept-only model) including an additive random term with the function *re()* for relating the random effects (time|tag_id) and the autocorrelation to the predictors. Models with different autocorrelation structures were compared by using Generalized Akaike information criterion (GAIC), which includes a k penalization for each degree of freedom of the model given by GAIC = -2$\hat{\mathcal{l}}$ + k × df, where $\hat{\mathcal{l}}$is the maximized log-likelihood and -2$\hat{\mathcal{l}}$ represents the global fitted deviance (GDEV) (Rigby and Stasinopoulos 2005). Starting from the model with the chosen additive random term (μ) we fitted additional models by consecutively incorporating the random structure to sub-models σ, ν and τ. We then used the function drop1() which, by using generalized likelihood ratio test (GLRT) to calculate the significance of dropping a term from the model, determined whether including the random effect was necessary for each parameter of the distribution.

Fitting a full model (sub-models μ, σ, ν and τ)

Following selection of the preferred random term, we fitted a linear additive model for the parameter μ by adding the covariates dH-KUD, dV-KS, body size and time × Lake. From this model, we built sets of candidate models by incorporating different non-linear smoothing functions (h_jk_) to the continuous predictors. These functions use a penalization to find the optimal value for the smoothing parameter or hyperparameter (λ) determining the degree of smoothness of the fitted curve between the smooth term and the predictor, which can be estimated by calculating the effective degrees of freedom (*edf*). All models were fitted using penalized P-splines with functions *ps()*, *pb()* and *pbz()*, that differ in how they optimize the smoothing parameter; *ps()* takes three degrees of freedom by default unless specified differently, which involves testing different values and re-fitting the model to find the optimal value; *pb()* automatically selects the smoothing parameter and thus it is not necessary to specify *edf*; and *pbz()* is based on the former function and is helpful for achieving more precise coefficients estimation (the fitted curve is shrinked toward zero degrees of freedom) (for more details on smoothing functions ^11^) To choose the one which minimized the GAIC criterion, we looked at the partial effects plots of predictors and re-fitted the model without the predictor of interest if necessary. After selection of the best full model (i.e., ‘mu’ sub-model from the parameter μ), we added all continuous predictors fitted with the preferred smoothing additive term to every other parameter of the distribution. We updated the mean base model by sequentially fitting extra predictors for each of σ, ν and τ, therefore building models with increasing complexity. To estimate the smoothing parameters, models were compared to minimize the values of GDEV and GAIC, and by visually checking the assumption of normality of the residuals with worm plots, i.e., detrended normal Q-Q plots of the (normalized quantile) residuals.

Fitting a final model on [0,1]

Once we had selected the final model, with general formula given by Equation 1_1-4_, we used the functions *term.plot()* and *getSmo()* to evaluate the influence of the predictors on each of the modelled components and to obtain details of the smooth terms. To further determine the impact of smooth functions on the final model and if a simplified model formula could be used, we computed GLRTs (see above) sequentially through each of four distribution parameters using the function drop1(). This allowed us to evaluate changes in the GAIC criterion of the model and calculate the statistical significance (p-value) of each of its terms. Note that this was only used to discern whether to add an additive term to a predictor but not to rule out the predictor, given the inclusion of a random variable as noted above. The final model could therefore include smoothing functions to all, some or none terms of the model and in the latter case the model would be re-fitted with linear terms.

Testing of SCI differences between lakes

Permutation inference were used to test differences among lakes and macrophytes sampling sessions (June, September) in the SCI. SCI was dependent variable while depth, transect and sampling season were used as explanatory variables. For comparison of groups of function, we used an $F$-max method ^12^, where $F$ statistic is computed pointwise and then the maximum over whole range of function is computed. The significance of the $F$-max statistic is determined by permutation method which have no assumptions for the residual distribution. The significance of the differences was computed from 1000 permutation of functions in the groups.

#### Extended Results

Effect of temperature and time to behavioural parameters

The extent of explored horizontal area (dH-KUD) and horizontal activity (HA) significantly decreased with temperature with similar trend in both lakes (Figs. S3 b, d). Temporal development of both parameters was differed between lakes as both decreased in LSC lake while increased in HSC lake (Figs. S3 a, c). Vertical utilization distribution (dV-KS) and vertical activity (VA) similarly decreased with temperature (Figs. S3 f, h) but showed opposite temporal pattern than dH-KUD and HA. dV-KS and VA increased during study period in LSC lake but slightly decreased in HSC lake (Figs. S3 e, g). Mean daily depth had similar pattern in both lakes with decrease of utilize depth with increasing temperature and during study period (Fig. S3 i, j).

GAMLSS model of pelagic habitat use (TOW)

The sample proportions of time spent in open water (TOW) were 0.55 and 0.005, with fish not using that particular habitat (i.e., 0%) 55% of the observed time and fully using it (i.e., 100%) in 0.005% of the cases, respectively. The model selection procedure yielded numerous candidate models varying in their smoothing functions as wells as the specific predictors fitted for each distribution parameter. Overall, there were issues of convergence as models increased in complexity across the four distribution parameters, but this mostly occurred at τ if the model included one or more continuous predictors along with the full additive random term. Dropping the slope of time solved these issues in some cases meaning that for these models we couldn’t fully address the relationship between the temporal variability of individuals as TOW was highest, i.e., when P(TOW) = 1 = p_1_. Convergence was generally achieved in models using the smoothing function *pb()* with better GDEV and GAIC values on average than models fitted with *ps(),* that can be explained by the automatic optimization of the smoothing parameter with the former function. On the other hand, models using the function *pbz()* proved inadequate to the inclusion of the additive random term as convergence issues occurred at the simpler sub-models ‘mu’ and/or ‘sigma’. This helped us to choose the models with function *pb()* and further determine if the smooth term could be dropped.

In the Table A7 we see that models fitted with distribution *BEINF*(μ, σ, ν, τ) overlap, in terms of GAIC, most models that do not specifically fit the parameter τ. The five best models fitted at *BEINF*(μ, σ, ν) are nearly identical (Nu_1-5_) while subsequent models are very close except Nu_9_. Indeed, the analysis of the coefficient estimates suggest that the predictors time and Lake are contributing greatly to model the shape parameter (ν), as is shown by the impact of dropping them (GAIC = 216.162) from the worst ranked model (GAIC = 192.825). It should be noted that the interaction time × Lake was highly significant for the mean parameter (μ), and therefore it was included at σ in order to reliably estimate the variance of the distribution as a function of its mean. Also note that overall, by accounting for heterogeneity in the variance, the complexity of sub-model ‘mu’ is decreased, i.e., the degrees of freedom drop from the ‘sigma’ sub-model. On the other hand, dH-KUD was important to model the distribution parameters μ, σ and ν, but not τ as all ‘Tau’ models excluded that predictor. Taking the first model Nu_1_ as the reference, the addition of one of either dV-KS or body size, doesn’t seem to improve the model fit and the simpler model could probably be retained with some certainty. Yet, given the minor differences in GDEV and GAIC we can say with confidence that the first five models are equally plausible. Comparing the degree of linearity, overall Nu_4_ and Nu_5_ model the parameter ν more non-linearly than Nu_1-3_ as reflected by their higher degrees of freedom (52.76 and 52.80 vs. 51.77, 52.02 and 52.07). The distribution of the residuals helped us to choose between these models.

The Q-Q plot in the Figure S4 shows that model Nu_4_ (bottom left) is the only to have all the observations with residuals within the 95% confidence interval (dotted elliptic curves), Nu_3_ (top right) follows closely with only one point outside the acceptance region, while Nu_1_ (top left), Nu_2_ (top center) and Nu_5_ (bottom center) appear to fit significantly worse than those two models. As there is little difference between Nu_3_ and Nu_4_ let’s keep both as the basis for further exploring the fitting of parameter τ.

According to the fitted predictors at *BEINF*(μ, σ, ν, τ), the addition of dH-KUD and dV-KS did not provide information to fit τ as models including one or both predictors stayed out of the candidate set, while body size appeared only in the ninth ranked model (Tau_9_). Overall, the addition of both time and Lake, either separately or as an interaction term, largely contributed to modeling τ, with models Tau_1_, Tau_2,_ Tau_3_ and Tau_4_ falling apart from the others in terms of GDEV and GAIC, and only the first two within an optimal range of 2 ∆GAIC. By excluding the slope of time from the additive random term, these models do not account for the individual variability of temporal trends and they rather evaluate the mean between-lakes difference with change over time (Tau_3_, Tau_4_) or not (Tau_1_, Tau_2_) as the probability of TOW is one. In addition, as with the other ‘Tau’ models, they also differ on which one of dV-KS or body size is used to model the probability at zero (ν). Following the first four models, the parameter τ appears to be better modeled by dropping the non-linear interaction and including a random slope for time that provides information on the temporal variation occurring at the individual level. This results in the shape parameter (τ) being modeled with a lower degree of non-linearity as evidenced by the reduction in the degrees of freedom from Tau_4_ (df (τ) = 26.76) to Tau_5_ (df (τ) = 12.86),

Models Tau_5_ and Tau_6_ are partly equivalent to models Nu_4_ and Nu_5_, as all four models fit a single intercept with zero degrees of freedom at τ, and therefore the probability at one (p_1_) is constant and independent of the predictors, i.e., none are used to model the parameter τ. When this occurs, σ is nearly equal to the coefficient of variation (CV), which may be subject to the effects of predictors at that distribution parameter. For example, the positive coefficient estimate for dH-KUD at σ suggests that, keeping other predictors constant, the range of TOW values is more heterogeneous as more horizontal area is utilized (i.e., CV significantly increases as the predictor increases), While this is true for ‘Nu’ models, ‘Tau’ models include the additive random term and thus they additionally account for the variance at τ, meaning that σ is no longer equivalent to the coefficient of variation. Note, however, that the latter have indeed lower GDEV and GAIC values indicating that they are qualitatively better for estimating the probability at one through the directed parameter formulation. Also noteworthy is the impact that the random slope of time has on the fitting of the parameter τ, as is shown by its removal from the ‘Nu’ sub-model, which leads to an increase in the GAIC criterion from Tau_5_ (185.93) to Tau_21_ (231.014). In the latter model the overall degrees of freedom for μ, σ and ν are lower than for any of the ‘Tau’ models, indicating that by including time as a linear predictor it is modeled with a lower degree of complexity at those distribution parameters; the degrees of freedom for τ are, however, high owing to the inclusion of the slope of time in the additive random term. The next best models (Tau_7_, Tau_8_)account for the mean between-lake differences in TOW but as long as time remains uncontrolled, the biological meaning disappears behind a high p-value for the factor Lake. Overall, although the two first models are apparently better, the third and fourth models have slightly better values of global deviance, which lead us to further compare the distribution of their residuals. This also gives us the opportunity to investigate the result of fitting the time by lake interaction or their main effects only.

In the Figure S5, we see that the distribution of the residuals is reasonably good in three of the top four ranked models. In all models several points partially fall outside the acceptance region between the two elliptic curves, however, models Tau_1_ (top left) and Tau_4_ (bottom right) in general appear to fit the distribution better than Tau_2_ (top right) and Tau_3_ (bottom left). In Tau_3_ one point lays clearly outside the lower confidence band, and in Tau_2_ several points are completely within the upper interval. This result proves it difficult to make a choice between the main-effects model Tau_1_ and the interaction model Tau_4_. Following the GAIC criterion and taking into account the high p-value of the interaction at τ in the second model we decided to finally keep the simpler first model as the most biologically relevant to further explore the relationship (fitted curve) between TOW and the predictors.

Finally, we explored if the selected model Tau_1_ (GAIC = 173.97) could be further simplified by removing some, all or none of the non-linear smoothing functions. First, the estimated total degrees of freedom for smoothing are very high for all sub-models reflecting a significant departure from linearity (i.e., two degrees of freedom) and indicating that non-linear models are required for each distribution parameter. Whether or not these non-linear sub-models could be further simplified depended on the effective degrees of freedom (*edf*) of the smoothing terms. Overall, the smooth terms for time and dV-KS had degrees of freedom relatively high (*edf* >10) and thus they were always included. For dH-KUD, *edf* were 6.13 ≈ 6, 4.34 ≈ 4 and 4.04 ≈ 4 for μ, σ and τ respectively, which are close to the degrees of freedom of a linear term especially for the ‘sigma’ and ‘nu’ sub-models. For body size, *edf* was 2.0004 ≈ 2 which corresponds to a linear tern and thus the smooth term was not required. Note that these values already include the two degrees of freedom from the fitting of the constant and the linear term to the model. To determine if the smoothing terms for dH-KUD could be dropped, we fitted additional models by excluding them one by one from each sub-model, with a special focus on the parameters σ and ν, given its proximity to linearity. Two of the new fitted models were very close in terms of GAIC criterion, one with only a smooth term for dH-KUD at ν (Tau_11_, GAIC = 171.80) and another with smooth terms for dH-KUD at μ and ν (Tau_12_, GAIC = 172.51). Following were a model with none smooth terms for dH-KUD (Tau_13_, GAIC = 175.02), with a single smooth term for dH-KUD at μ (Tau_14_, GAIC = 175.73) or at σ (Tau_15_, GAIC = 176.42). According to GAIC, we kept the first two models and next we checked the distribution of their residuals which showed that model Tau_12_ with two smooth terms at μ and ν was preferred.

Therefore, model Tau_12_ was a simplified form of Tau_1_ without the smoothing term for body size from parameter ν and two relevant smoothing terms fitted for dH-KUD at μ and ν, Finally, the model was re-fitted using the *edf* for each smooth term which resulted in an improved distribution of residuals that minimized the global deviance and captured better the shape of the curve at zero and one. The final model can be expressed with the following notation (see Equation 4):

$$\mathrm{TOW}=\left[ \begin{aligned} \mathrm{logit}\left( \mu\right)=\alpha_{0}+ pb\left( \mathrm{time} \right)\times Lake + pb(dHKUD)+pb\left( \mathrm{dVKS} \right)+re\left( \sim time | ID, corARMA \right) \\ \mathrm{logit}\left( \sigma\right)=\beta_{0}+ pb\left( \mathrm{time} \right)\times Lake + dHKUD+pb\left( \mathrm{dVKS} \right)+re\left( \sim time | ID, corARMA \right) \\ \log\left( \nu\right)= \delta_{0}+ pb\left( \mathrm{time} \right)+ Lake + pb\left( \mathrm{dHKUD} \right)+Body size+re\left( \sim time | ID, corARMA \right) \\ \log\left( \tau\right)=\rho_{0} + pb\left( \mathrm{time} \right)+ Lake + re\left( \sim1 | ID, corARMA \right) \end{aligned} \right]$$

, where $\mathrm{TOW}\sim BEINF\left( \mu,\sigma, \nu, \tau\right)$ with three P-splines smoothing terms for parameter μ, two for parameters σ and ν, and a single smoothing term for parameter τ. The first three sub-models fit a random slope-intercept with autocorrelation in the random additive term while sub-model τ fits a single random intercept with autocorrelation.

The model in R code is expressed as follow:

m_TOW_final<- gamlss(hab.prop ~ pb(time)*up_lake + pb(log_area95) + pb(k1size) +

re(random=~time|tag_id, method="REML",correlation=corARMA(q=1)),

sigma.formula=~pb(time)*up_lake + log_area95 + pb(k1size) +

re(random=~time|tag_id, method="REML", correlation=corARMA(q=1)),

nu.formula=~pb(time)+up_lake + pb(log_area95) + tl_mm +

re(random=~time|tag_id, method="REML", correlation=corARMA(q=1)),

tau.formula=~pb(time)+up_lake + re(random=~1|tag_id, method="REML",

correlation=corARMA(q=1)), family = BEINF, data = open_water_prop_s)

To calculate the probabilities at TOW=0 (p_0_) and TOW=1 (p_1_) from the final model we use the following code:

*# Lake p0*

coef.lake.nu<-coef(m_TOW_final, "nu")[3]

OR.lake.nu<-exp(coef.lake.nu)

OR.lake.nu.lwer<-exp(coef.lake.nu - 1.96 *0.140771)

OR.lake.nu.uper<-exp(coef.lake.nu + 1.96 *0.140771)

*# dH-KUD p0*

coef.log_area95.nu<-coef(m_TOW_final, "nu")[4]

OR.log_area95.nu<-exp(coef.log_area95.nu)

OR.log_area95.nu.lwer<-exp(coef.log_area95.nu - 1.96 *0.101915)

OR.log_area95.nu.uper<-exp(coef.log_area95.nu + 1.96 *0.101915)

*# tl_mm p0*

coef.tl_mm.nu<-coef(m_TOW_final, "nu")[5]

OR.tl_mm.nu<-exp(coef.tl_mm.nu)

OR.tl_mm.nu.lwer<-exp(coef.tl_mm.nu - 1.96 *0.068962)

OR.tl_mm.nu.uper<-exp(coef.tl_mm.nu + 1.96 *0.068962)

*# Lake p1*

coef.lake.tau<-coef(m_TOW_final, "tau")[3]

OR.lake.tau<-exp(coef.lake.tau)

OR.lake.tau.lwer<-exp(coef.lake.tau - 1.96 *1.11811)

OR.lake.tau.uper<-exp(coef.lake.tau + 1.96 *1.11811)

Stable isotopes analysis

Stable isotope analysis was done for prey species as well as for littoral and pelagic primary producers and/or consumers. Samples for the analysis were collected in years 2013 and 2014, further details on samples acquisition and analysis are given in ^13,14^. Biplots depicting the mean ± SD δ13C and δ15N values of pike (tracked during this study) and putative prey fishes, as well as all individual pike SIA values are given in Fig. S6. Such comparison showed that pike and prey fishes relied strongly on littoral benthic resource, with the δ13C values of fish being closer to those of littoral benthos as compared to low δ13C values of pelagic zooplankton. Coregonids (i.e., whitefish) in LSCL was the only prey fish species with δ13C values resembling more those of pelagic planktonic than of littoral benthic food resources. Hence, the pike individual in LSCL with an exceptionally low littoral reliance estimate (i.e., low δ13C value) has most likely substantially fed on pelagic zooplanktivorous Coregonids.

Analysis of growth rate using linear regression

Both dH-KUD and dV-KS were highly correlated with horizontal activity (r=0.85) and vertical activity (r=0.81) respectively, resulting in high multicollinearity where models were never selected. However, the RF analysis detected a meaningful interaction between those two variables. Adding the interaction to a model accounting for the differences between lakes and age (model 2, R^2^~ 0.60; Tab. A8) contributed to explain 10% higher variation in growth rate (model 8, R^2^~ 0.70) and marginally improved the model fit (Likelihood-ratio test, χ^2^_4,7_ = 7.08, *P* < 0.1) (Table A8). The interaction had a significant impact on growth (dH-KUD × dV-KS, t = 2.448, *P* = 0.025) but not their main effects, which is consistent with a crossover interaction and the lack of evidence for the inclusion of main effects separately (Likelihood-ratio test, χ^2^_6,7_ = 6.9, *P* < 0.01). Note that total significance was not observed probably due to the low sample size (n=24) with only three observations above the threshold of 1.32 for dV-KS.

#### References

1. Baktoft, H., Gjelland, K. Ø., Økland, F. & Thygesen, U. H. Positioning of aquatic animals based on time-of-arrival and random walk models using YAPS (Yet Another Positioning Solver). *Sci. Rep.* **7**, 1–10 (2017).

2. Gjelland, K. Ø. & Hedger, R. D. Environmental influence on transmitter detection probability in biotelemetry: developing a general model of acoustic transmission. *Methods Ecol. Evol.* **4**, 665–674 (2013).

3. Wood, S. N. Fast stable restricted maximum likelihood and marginal likelihood estimation of semiparametric generalized linear models. *J. R. Stat. Soc. Ser. B Stat. Methodol.* **73**, 3–36 (2011).

4. Boulesteix, A. L., Janitza, S., Kruppa, J. & König, I. R. Overview of random forest methodology and practical guidance with emphasis on computational biology and bioinformatics. *Wiley Interdiscip. Rev. Data Min. Knowl. Discov.* **2**, 493–507 (2012).

5. Biau, G. & Scornet, E. A random forest guided tour. *Test* **25**, 197–227 (2016).

6. Cutler, D. R. *et al.* Random forests for classification in ecology. *Ecology* **88**, 2783–2792 (2007).

7. Zeileis, A. & Hothorn, T. Diagnostic Checking in Regression Relationships. *R news* **2**, 7–10 (2002).

8. Lenth, R. emmeans: Estimated Marginal Means, aka Least-Squares Means. R package version 1.5.1. (2020).

9. Stasinopoulos, D. M. & Rigby, R. A. Generalized additive models for Location Scale and Shape (GAMLSS) in R. (2007) doi:10.18637/jss.v023.i07.

10. Rigby, R. A. & Stasinopoulos, D. M. *A Flexible Regression Approach Using GAMLSS in R*. (London Metropolitan University, 2009).

11. Stasinopoulos, D. M., Rigby, R. A., Heller, Z. G., Voudouris, V. & De Bastiani, F. *Flexible Regression and Smoothing Using GAMLSS in R*. (CRC Press, 2017).

12. Winkler, A. M., Ridgway, G. R., Webster, M. A., Smith, S. M. & Nichols, T. E. Permutation inference for the general linear model. *Neuroimage* **92**, 381–397 (2014).

13. Vejříková, I. *et al.* Macrophytes shape trophic niche variation among generalist fishes. *PLoS One* **12**, 1–13 (2017).

14. Eloranta, A. P. *et al.* Some like it deep: Intraspecific niche segregation in ruffe (Gymnocephalus cernua). *Freshw. Biol.* **62**, 1401–1409 (2017).

# Tables

Table A1. Number and mean weight of stocked pike individuals into studies lakes

| Lake | Year | Mean weight (g) | Number |
| --- | --- | --- | --- |
| HSC | 2003 | 1.7 | 8500 |
|  | 2005 | 303 | 789 |
| LSC | 2011 | 1165 | 984 |
|  | 2012 | 1449 | 374 |
|  | 2013 | 885 | 974 |

TABLE A2-A6. Selection of Linear mixed-effects models (LMMs) analysing the effects of phenotype (body length) and environmental factors (water temperature) on differences in pike behaviour across two lakes (low structural complexity lake -LSC set as the reference): Horizontal area use (H-KUD, A2), vertical range use (V-KS, A3), depth (A4), horizontal activity (A5) and vertical activity (A5). Candidate models are ranked from more to less parsimonious according to Akaike information criterion with finite size correction (AICc). In italics, models representing the 95% confidence set (model probabilities sum to > 0.95). In bold, the final best fit model selected. A log-likelihood ratio test (LRT) was additionally used between each two nested models to test alternative hypothesis. Numbers refer to β standardized estimates (mean-cantered and scaled by 2 s.d.) and ± 95% confidence intervals (in parenthesis); the response remains untransformed. All variables were modelled according to an ARMA autocorrelation structure of order (p=1, q=1). For simplicity random effects and ARMA parameters are omitted.

*χ^2^ _LRT_*, Chi-squared of the LRT between models pairs (significant tests were set at the 0.95 –α level); *R^2^_m_*, marginal r-squared reflecting the proportion of variation explained by fixed effects; *R^2^_c_*, conditional r-squared indicating the proportion of the variance explained by fixed and random effects*; AICc*, corrected Akaike’s information criterion for small sample size; *ki*, number of parameters in the model; *∆AIC*, difference in AICc value relative to the model with lowest *AICc*. Differences <2 in AICc indicate minor (not relevant) differences between models; *wi*, weight of evidence in support of a model as being the best fit (range 0-1); *∑wi*, cumulative sum of weights (range 0-1); *logLik*, Log-likelihood estimation of the model computed by Maximum Likelihood (ML).

Significance p-values for the regression estimates (via Wald-statistics) and LRTs: ^*^p<0.1; ^**^p<0.05; ^***^p<0.01

| Table A2. LMMs selection table for dH-KUD | | | | | | |
| --- | --- | --- | --- | --- | --- | --- |
|  | | | | | | |
|  | *Dependent variable:* dH-KUD [log(m^2^)] | | | | | |
|  |  | | | | | |
|  | Model 1 | Model 2 | Model 3 | Model 4 | Model 5 | *Model 6* |
| (Intercept) | 0.713^***^  [0.511–0.915] | 0.814^***^  [0.722–0.906] | 0.804^***^  [0.711–0.897] | 0.806^***^  [0.641–0.971] | 0.807^***^  [0.642–0.972] | 0.772^***^  [0.625–0.920] |
| time × lake | -0.003^*^  [-0.005–0.0002] | -0.003^**^  [-0.005–-0.001] | -0.003^**^  [-0.005–-0.001] | -0.003^*^  [-0.005–0.00002] | -0.003^*^  [-0.005–0.0001] | -0.003^*^  [-0.005–0.00002] |
| time | 0.002  [-0.0004–0.003] | 0.002^**^  [0.00003–0.003] | 0.002^**^  [0.0002–0.003] | 0.002^*^  [-0.0002–0.004] | 0.002^*^  [-0.0003–0.004] | 0.002^*^  [-0.0003–0.004] |
| lake | 0.567^***^  [0.286–0.848] | 0.420^***^  [0.287–0.553] | 0.412^***^  [0.278–0.546] | 0.406^***^  [0.173–0.640] | 0.404^***^  [0.171–0.637] | 0.390^***^  [0.183–0.598] |
| water temperature × lake |  |  |  |  | -0.014  [-0.061–0.033] |  |
| water temperature |  |  | -0.041^***^  [-0.069–-0.014] | -0.027^**^  [-0.049–-0.006] | -0.020  [-0.053–0.013] | -0.027^**^  [-0.048–-0.005] |
| body length × lake |  |  |  |  |  | 0.235^***^  [0.106–0.365] |
| body length |  | 0.213^***^  [0.160–0.266] | 0.212^***^  [0.160–0.265] | 0.209^***^  [0.136–0.283] | 0.209^***^  [0.135–0.282] | 0.135^***^  [0.062–0.208] |
| water temperature × body length |  |  |  | 0.019^*^  [-0.003–0.042] | 0.022^*^  [-0.002–0.046] | 0.020^*^  [-0.002–0.042] |
| χ^2^ _LRT_ | - | 24.55^***^ | 8.90^***^ | 44.46^***^ | 0.36 | 10.16^***^ |
| R^2^_m_/R^2^_c_ | 0.21/0.60 | 0.39/0.49 | 0.39/0.49 | 0.38/0.59 | 0.38/0.49 | 0.43/0.60 |
| *k_i_* | 10 | 11 | 12 | 13 | 14 | 14 |
| AICc | 95.26 | 121.83 | 114.97 | 72.53 | 74.2 | 64.4 |
| ∆AICc | 30.86 | 57.43 | 50.56 | 8.13 | 9.8 | 0 |
| *w_i_* | 0 | 0 | 0 | 0.02 | 0.01 | 0.98 |
| *∑wi* | 1 | 1 | 1 | 0.99 | 1 | 0.98 |
| logLik | -37.57 | -49.85 | -45.4 | -23.17 | -22.99 | -18.09 |

| Table A3. LMMs selection table for dV-KS | | | | | |
| --- | --- | --- | --- | --- | --- |
|  | | | | | |
|  | *Dependent variable:* dV-KS [log(m)] | | | | |
|  |  | | | | |
|  |  | | | | |
|  | Model 1 | Model 2 | *Model 3* | *Model 4* | *Model 5* |
|  | | | | | |
| (Intercept) | 1.206^***^  [1.068–1.344] | 1.150^***^  [0.746–1.555] | 1.536^***^  [1.273–1.799] | 1.354^***^  [1.005–1.702] | 1.385^*^  [-0.070–2.840] |
| time × lake | 0.002  [-0.002–0.005] | 0.002  [-0.002–0.005] | 0.002  [-0.002–0.005] | 0.002  [-0.002–0.005] | 0.002  [-0.002–0.005] |
| time | -0.001  [-0.003–0.002] | -0.001  [-0.003–0.002] | -0.001  [-0.003–0.002] | -0.001  [-0.003–0.002] | -0.001  [-0.003–0.002] |
| lake | 0.114  [-0.080–0.309] | 0.107  [-0.092–0.306] | 0.11  [-0.082–0.303] | 0.464^*^  [-0.020–0.949] | 0.102  [-0.094–0.299] |
| water temperature × lake |  |  |  | -0.017  [-0.039–0.004] |  |
| water temperature |  |  | -0.016^***^  [-0.027–-0.005] | -0.007  [-0.023–0.008] | -0.012  [-0.079–0.056] |
| water temperature × body length |  |  |  |  | -0.00001  [-0.0001–0.0001] |
| body length |  | 0.0001  [-0.0004–0.001] |  |  | 0.0002  [-0.002–0.002] |
| χ^2^ _LRT_ | - | 0.07 | 8.23^***^ | 2.41 | 0.10 |
| R^2^_m_/R^2^_c_ | 0.06/0.29 | 0.06/0.29 | 0.07/0.29 | 0.07/0.29 | 0.07/0.3 |
| *k_i_* | 10 | 11 | 11 | 12 | 13 |
| AICc | 1982.15 | 1984.1 | 1975.94 | 1975.55 | 1979.89 |
| ∆AICc | 6.6 | 8.55 | 0.39 | 0 | 4.34 |
| *w_i_* | 0.02 | 0.01 | 0.41 | 0.5 | 0.06 |
| *∑wi* | 0.99 | 1 | 0.92 | 0.5 | 0.97 |
| logLik | -981.02 | -980.98 | -976.9 | -975.69 | -976.85 |

| Table A4. LMMs selection table for mean depth | | | | | |
| --- | --- | --- | --- | --- | --- |
|  | | | | | |
|  | Dependent variable: Depth [log(m)] | | | | |
|  |  | | | | |
|  |  | | | | |
|  | Model 1 | Model 2 | Model 3 | Model 4 | Model 5 |
|  | | | | | |
| (Intercept) | 1.374***  [1.099–1.648] | 1.464***  [1.197–1.731] | 1.474***  [1.201–1.747] | 1.474***  [1.201–1.747] | 1.384***  [1.102–1.665] |
| time × lake | 0.002  [-0.002–0.006] | 0.002  [-0.002–0.006] | 0.002  [-0.002–0.006] | 0.002  [-0.002–0.006] | 0.002  [-0.002–0.006] |
| time | 0.004^***^  [0.001–0.007] | 0.004^***^  [0.001–0.007] | 0.004^***^  [0.001–0.007] | 0.004^***^  [0.001–0.007] | 0.004^***^  [0.001–0.007] |
| lake | -0.065  [-0.448–0.319] | -0.216  [-0.596–0.163] | -0.212  [-0.600–0.175] | -0.213  [-0.600–0.174] | -0.061  [-0.455–0.332] |
| water temperature |  |  | 0.050^**^  [0.004–0.095] | 0.049^**^  [0.004–0.095] | 0.050^**^  [0.005–0.095] |
| water temperature × body length |  |  |  | -0.004  [-0.050–0.043] |  |
| body length |  | 0.194^**^  [0.051–0.338] | 0.194^**^  [0.049–0.339] | 0.194^**^  [0.048–0.339] |  |
| χ^2^ _LRT_ | - | 6.23^**^ | 4.40^**^ | 0.02 | 6.12^**^ |
| R^2^_m_/R^2^_c_ | 0.08/0.41 | 0.14/0.42 | 0.15/0.43 | 0.15/0.43 | 0.09/0.43 |
| *k_i_* | 10 | 11 | 12 | 13 | 11 |
| AICc | 1867.8 | 1863.59 | 1861.21 | 1863.22 | 1865.31 |
| ∆AICc | 6.59 | 2.38 | 0 | 2 | 4.09 |
| *w_i_* | 0.02 | 0.17 | 0.54 | 0.2 | 0.07 |
| *∑wi* | 1 | 0.91 | 0.54 | 0.74 | 0.98 |
| logLik | -923.84 | -920.73 | -918.52 | -918.51 | -921.58 |

| Table A5. LMMs selection table for horizontal activity | | | | | | | | | | |
| --- | --- | --- | --- | --- | --- | --- | --- | --- | --- | --- |
|  | | | | | | | | | | |
|  | | Dependent variable: Horizontal activity [sqrt(m/s-1)] | | | | | | | | |
|  |  |  | | | | | | | | |
|  |  | Model 1 | | Model 2 | | Model 3 | | Model 4 | | Model 5 |
|  | | | | | | | | | | |
| (Intercept) | | 0.043***  [0.032–0.054] | | 0.046***  [0.036–0.057] | | 0.045***  [0.035–0.056] | | 0.045***  [0.035–0.056] | | 0.045***  [0.035–0.056] |
| time × lake | | -0.0002^**^  [-0.0004–-0.00004] | | -0.0002^**^  [-0.0004–-0.00004] | | -0.0002^**^  [-0.0004–-0.00004] | | -0.0002^**^  [-0.0004–-0.00005] | | -0.0002^**^  [-0.0004–-0.00005] |
| time | | 0.00002  [-0.0001–0.0001] | | 0.00002  [-0.0001–0.0001] | | 0.00003  [-0.0001–0.0001] | | 0.00003  [-0.0001–0.0001] | | 0.00003  [-0.0001–0.0002] |
| lake | | 0.035^***^  [0.020–0.050] | | 0.031^***^  [0.016–0.046] | | 0.030^***^  [0.016–0.045] | | 0.031^***^  [0.016–0.045] | | 0.031^***^  [0.016–0.045] |
| water temperature × lake | |  | |  | |  | |  | | 0.001  [-0.002–0.005] |
| water temperature | |  | |  | | -0.003^***^  [-0.004–-0.001] | | -0.003^***^  [-0.004–-0.001] | | -0.003^***^  [-0.006–-0.001] |
| water temperature × body length | |  | |  | |  | | 0.001  [-0.0003–0.003] | |  |
| body length | |  | | 0.006^**^  [0.001–0.011] | | 0.006^**^  [0.001–0.011] | | 0.006^**^  [0.001–0.011] | | 0.006^**^  [0.001–0.011] |
|  | | | | | | | | | | |
| χ^2^ _LRT_ | - | | 4.78^**^ | | 11.37^***^ | | 2.52 | | 0.88 | |
| R^2^_m_/R^2^_c_ | 0.19/0.47 | | 0.22/0.47 | | 0.23/0.47 | | 0.23/0.48 | | 0.23/0.47 | |
| *k_i_* | 10 | | 11 | | 12 | | 13 | | 13 | |
| AICc | -10361.62 | | -10364.37 | | -10373.72 | | -10374.22 | | -10372.57 | |
| ∆AICc | 12.6 | | 9.85 | | 0.5 | | 0 | | 1.65 | |
| *w_i_* | 0 | | 0 | | 0.35 | | 0.45 | | 0.2 | |
| *∑wi* | 1 | | 1 | | 0.8 | | 0.45 | | 1 | |
| logLik | 5190.87 | | 5193.25 | | 5198.94 | | 5200.2 | | 5199.38 | |

| Table A6. LMMs selection table for vertical activity | | | | | |
| --- | --- | --- | --- | --- | --- |
|  | | | | | |
|  | Dependent variable: Vertical activity [sqrt(m/s-1)] | | | | |
|  |  | | | | |
|  |  | | | | |
|  | Model 1 | Model 2 | Model 3 | Model 4 | Model 5 |
|  | | | | | |
| (Intercept) | 0.005***  [0.004–0.006] | 0.005***  [0.004–0.006] | 0.005***  [0.004–0.006] | 0.005***  [0.005–0.006] | 0.005***  [0.005–0.005] |
| time × lake | 0.00001  [-0.00001–0.00003] | 0.00001  [-0.00001–0.00003] | 0.00001  [-0.00001–0.00003] | 0.00001  [-0.00000–0.00002] | 0.00001  [-0.00000–0.00002] |
| time | 0  [-0.00001–0.00001] | 0  [-0.00001–0.00001] | 0  [-0.00001–0.00001] | 0  [-0.00001–0.00001] | 0  [-0.00001–0.00001] |
| lake | 0.001  [-0.001–0.002] | 0.001  [-0.0004–0.002] | 0.001  [-0.001–0.002] | 0.001^*^  [-0.0001–0.001] | 0.001^*^  [-0.00005–0.001] |
| water temperature × lake |  |  |  | -0.0003^*^  [-0.001–0.00001] | -0.0003^*^  [-0.001–0.00001] |
| water temperature |  |  | -0.0002^***^  [-0.0003–-0.0001] | -0.0001  [-0.0003–0.0001] | -0.0001  [-0.0003–0.0001] |
| body length × lake |  |  |  |  | 0.001^*^  [-0.00001–0.001] |
| body length |  | -0.0003  [-0.001–0.0002] |  |  | -0.0004^**^  [-0.001–-0.00002] |
| χ^2^ _LRT_ | - | 1.46 | 9.82^***^ | 13.47^***^ | 5.24^*^ |
| R^2^_m_/R^2^_c_ | 0.07/0.40 | 0.09/0.40 | 0.08/0.40 | 0.09/0.35 | 0.11/0.37 |
| *k_i_* | 11 | 10 | 11 | 14 | 12 |
| AICc | -19087.15 | -19079.35 | -19078.79 | -19072.84 | -19071.66 |
| ∆AICc | 0 | 7.79 | 8.36 | 14.31 | 15.49 |
| *w_i_* | 0.96 | 0.02 | 0.01 | 0 | 0 |
| *∑wi* | 0.96 | 0.98 | 1 | 1 | 1 |
| logLik | 9554.64 | 9549.73 | 9550.46 | 9550.53 | 9547.91 |

TABLE [A7.](https://www.ncbi.nlm.nih.gov/pmc/articles/PMC5911484/table/T5/) GAMLSS models analysing the effects of phenotype (body length) and behavior (H-KUD, V-KS) of pike on the pelagic habitat use as a function of the time spent in open water (TOW). All models were fitted according to a zero-one inflated beta distribution (BEINF in R package gamlss) and ordered from best to worse fitting according to their global fitted deviance (GDEV) and GAIC criterion. df, the degrees of freedom used for the four distribution parameters μ, σ, ν and τ and the total number of degrees of freedom of the models; t refers to the continuous variable ‘time’ included as a fixed effect; in the additive random term ‘time’ was included as a random slope (tslope), otherwise the model was fitted with only a random-intercept (tag_ID) by default (i.e., unspecified). L, categorical variable lake with two levels (HSC, LSC); H, -KUD; V, V-KS); pseudo-R2, generalized pseudo R-squared (Nagelkerke 1991).

| Model | Description | df (μ) | df (σ) | df (ν) | df (τ) | df | GDEV | GAIC |
| --- | --- | --- | --- | --- | --- | --- | --- | --- |
|  |  |  |  |  |  |  |  |  |
|  | BEINF(μ, σ, ν) |  |  |  |  |  |  |  |
|  |  |  |  |  |  |  |  |  |
| Nu1 | ν (th × L, Hh, tslope) | 71.94 | 75.20 | 51.77 | 1 | 199.91 | -210.80 | 189.014 |
| Nu2 | ν (th, L, Hh, Vh, tslope) | 71.94 | 75.20 | 52.02 | 1 | 200.16 | -211.29 | 189.039 |
| Nu3 | ν (th, L, Hh, BSh, tslope) | 71.94 | 75.20 | 52.07 | 1 | 200.21 | -210.74 | 189.674 |
| Nu4 | ν (th × L, Hh, Vh, tslope) | 71.94 | 75.20 | 52.76 | 1 | 200.89 | -211.59 | 190.208 |
| Nu5 | ν (th × L, Hh, BSh, tslope) | 71.94 | 75.20 | 52.80 | 1 | 200.94 | -211.00 | 190.883 |
| Nu6 | ν (th × L, Hh, Vh, BSh, tslope) | 71.94 | 75.20 | 53.80 | 1 | 201.94 | -211.81 | 192.073 |
| Nu7 | ν (th × L, Hh, Vh, tslope) | 72.94 | 75.11 | 52.76 | 1 | 201.82 | -211.48 | 192.150 |
| Nu8 | ν (th × L, Hh, BSh, tslope) | 72.94 | 75.11 | 52.80 | 1 | 201.86 | -210.90 | 192.825 |
| Nu9 | ν (Hh, Vh, tslope) | 71.94 | 75.20 | 46.21 | 1 | 194.35 | -172.54 | 216.162 |
|  |  |  |  |  |  |  |  |  |
|  | BEINF(μ, σ, ν, τ) |  |  |  |  |  |  |  |
|  |  |  |  |  |  |  |  |  |
| Tau1 | Nu3~τ (th, L) | 71.94 | 75.20 | 52.27 | 25.69 | 225.09 | -276.22 | 173.973 |
| Tau2 | Nu5~τ (th, L) | 71.94 | 75.20 | 52.98 | 25.71 | 225.84 | -276.71 | 174.965 |
| Tau3 | Nu4~τ (th × L) | 71.94 | 75.20 | 52.97 | 26.79 | 226.89 | -277.65 | 176.144 |
| Tau4 | Nu5~τ (th × L) | 71.94 | 75.20 | 52.99 | 26.76 | 226.89 | -276.81 | 176.963 |
| Tau5 | Nu4~τ (1, tslope) | 71.94 | 75.20 | 52.71 | 12.82 | 212.67 | -239.41 | 185.926 |
| Tau6 | Nu5~τ (1, tslope) | 71.94 | 75.20 | 52.75 | 12.86 | 212.75 | -238.84 | 186.660 |
| Tau7 | Nu4~τ (L, tslope) | 71.94 | 75.20 | 52.71 | 13.88 | 213.73 | -239.47 | 187.990 |
| Tau8 | Nu5~τ (L, tslope) | 71.94 | 75.20 | 52.75 | 13.93 | 213.82 | -238.91 | 188.732 |
| Tau9 | Nu4~τ (BSh) | 71.94 | 75.20 | 52.76 | 2.92 | 202.83 | -215.95 | 189.701 |
| Tau10 | Nu4~τ (t × L, tslope) | 71.94 | 75.20 | 52.69 | 14.89 | 214.71 | -239.53 | 189.903 |
| Tau11 | Nu4~τ (t × L) | 71.94 | 75.20 | 52.69 | 14.89 | 214.71 | -239.53 | 189.904 |
| Tau12 | Nu2~τ (th, L) | 71.94 | 75.20 | 52.02 | 14.18 | 213.34 | -236.58 | 190.098 |
| Tau13 | Nu4(BS)~τ (1) | 71.94 | 75.20 | 52.76 | 1.041 | 200.94 | -211.59 | 190.290 |
| Tau14 | Nu4~τ (1) | 71.94 | 75.20 | 52.76 | 1.041 | 200.94 | -211.59 | 190.292 |
| Tau15 | Nu5~τ (BSh) | 71.94 | 75.20 | 52.81 | 2.964 | 202.911 | -215.49 | 190.329 |
| Tau16 | Nu5~τ (t × L, tslope) | 71.94 | 75.20 | 52.73 | 14.94 | 214.81 | -239.03 | 190.600 |
| Tau17 | Nu5~τ (t × L) | 71.94 | 75.20 | 52.73 | 14.94 | 214.81 | -239.03 | 190.601 |
| Tau18 | Nu4~τ (L) | 71.94 | 75.20 | 52.76 | 2.04 | 201.94 | -212.96 | 190.921 |
| Tau19 | Nu4~τ (th, L) | 71.94 | 75.20 | 52.73 | 14.22 | 214.09 | -237.02 | 191.159 |
| Tau20 | Nu4(edf )~τ (1) | 78.25 | 81.49 | 59.78 | 1.05 | 220.57 | -241.96 | 199.187 |
| Tau21 | Nu4(t)~τ (1, tslope) | 57.84 | 65.51 | 45.34 | 19.57 | 188.26 | -145.52 | 231.014 |

h The predictor was fitted with a penalized P-spline smoothing function hjk (see Equation 11-4). Otherwise, the linear term is shown.

| TABLE A8. Linear models analysing the effects of phenotype (body length) and behaviour (H-KUD, V-KS, horizontal activity, vertical activity) on pike growth rate expressed as body increment during last year prior to study (N = 24). In italics, models representing the 95% confidence set (model probabilities sum to > 0.95). In bold, the final model selected according to the model fit improvement as determined by log-likelihood ratio (χ2 LRT); R2, r-squared statistic measuring the proportion of variation in growth rate explained by fixed effects; Adjusted R2, r-squared adjusted for the number of predictors in the model; β standardized estimates (mean-cantered and scaled by 2 s.d.) and 95% confidence intervals (in parenthesis) are shown. Significance p-values for the regression estimates (via Wald-statistics) and LRTs: *p<0.1; **p<0.05; ***p<0.01 | | | | | | | | |
| --- | --- | --- | --- | --- | --- | --- | --- | --- |
|  | | | | | | | | |
|  | Model 1 | Model 2 | Model 3 | Model 4 | Model 5 | Model 6 | Model 7 | Model 8 |
|  | | | | | | | | |
| (Intercept) | 123.685*** | 103.918*** | 87.172 | 105.567*** | 105.605*** | 105.368*** | 109.605*** | 89.963*** |
|  | [104.611–142.759] | [77.066–130.769] | [-64.317–238.661] | [74.645–136.489] | [76.457–134.752] | [72.315–138.421] | [81.856–137.354] | [57.689–122.236] |
|  |  |  |  |  |  |  |  |  |
| age | -49.526*** | -45.798*** | -47.784*** | -46.503*** | -44.178*** | -46.341*** | -40.012*** | -41.188*** |
|  | [-69.011–-30.041] | [-64.541–-27.055] | [-73.865–-21.703] | [-66.618–-26.387] | [-65.534–-22.821] | [-66.727–-25.955] | [-60.382–-19.642] | [-61.860–-20.516] |
|  |  |  |  |  |  |  |  |  |
| lake |  | 36.493* | 33.384 | 33.449 | 33.379 | 33.816 | 25.994 | 52.272** |
|  |  | [-0.332–73.319] | [-13.370–80.138] | [-12.438–79.336] | [-8.403–75.162] | [-16.866–84.498] | [-13.492–65.480] | [4.784–99.760] |
|  |  |  |  |  |  |  |  |  |
| body length |  |  | 0.022 |  |  |  |  |  |
|  |  |  | [-0.176–0.220] |  |  |  |  |  |
|  |  |  |  |  |  |  |  |  |
| dH-KUD |  |  |  | 2.690 |  |  |  | 0.802 |
|  |  |  |  | [-20.439–25.819] |  |  |  | [-20.373–21.977] |
|  |  |  |  |  |  |  |  |  |
| dV-KS |  |  |  |  | 4.064 |  |  | -11.062 |
|  |  |  |  |  | [-19.639–27.768] |  |  | [-35.831–13.706] |
| horizontal activity |  |  |  |  |  | 2.017 |  |  |
|  |  |  |  |  |  | [-23.483–27.517] |  |  |
|  |  |  |  |  |  |  |  |  |
| vertical activity |  |  |  |  |  |  | 14.824 |  |
|  |  |  |  |  |  |  | [-7.385–37.032] |  |
|  |  |  |  |  |  |  |  |  |
| dH-KUD × dV-KS |  |  |  |  |  |  |  | 28.333** |
|  |  |  |  |  |  |  |  | [5.646–51.020] |
|  |  |  |  |  |  |  |  |  |
|  |  |  |  |  |  |  |  |  |
|  | | | | | | | | |
| χ2 LRT | - | 3.96** | 0.06 | 0.06 | 0.13 | 0.02 | 1.97 | 7.08* |
| R2/Adjusted R2 | 0.53/0.51 | 0.60/0.56 | 0.60/0.54 | 0.60/0.54 | 0.60/0.54 | 0.60/0.54 | 0.63/0.58 | 0.70/0.62 |
|  |  |  |  |  |  |  |  |  |
| ki | 3 | 4 | 5 | 5 | 5 | 5 | 5 | 7 |
| AICc | 258.71 | 257.65 | 260.82 | 260.82 | 260.75 | 260.85 | 258.91 | 261.47 |
| ∆AICc | 1.06 | 0 | 3.17 | 3.17 | 3.09 | 3.2 | 1.26 | 3.82 |
| wi | 0.19 | 0.32 | 0.07 | 0.07 | 0.07 | 0.07 | 0.17 | 0.05 |
| ∑wi | 0.51 | 0.32 | 0.89 | 0.82 | 0.75 | 0.95 | 0.69 | 1 |
| logLik | -125.76 | -123.77 | -123.75 | -123.74 | -123.71 | -123.76 | -122.79 | -120.23 |
|  | | | | | | | | |

# Figures


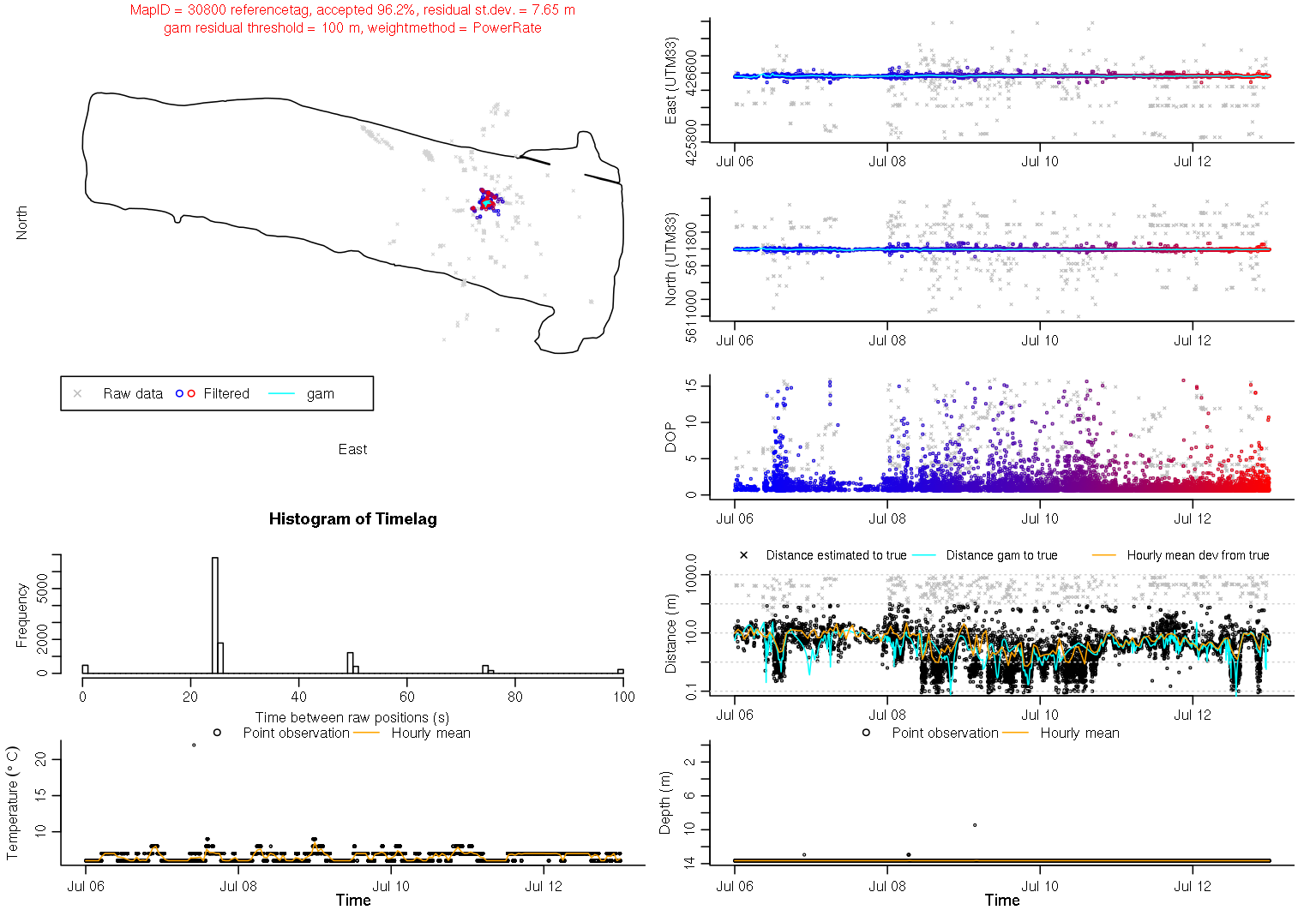


Figure S1. Example of filtering one week of position data for a reference tag. Grey colour indicates positions not accepted by the filtering algorithm, blue to red indicates accepted positions with colour indicating time; blue being oldest and red being latest positions. The cyan-coloured line indicates the final gam-smoothed track. a) Horizontal position estimates, with the outline of Lake Milada indicated by a black line, b) easting of positions versus time, c) northing of position versus time, d) dilution of precision (DOP), a precision measure produced by U-MAP indicating the effective precision of a position estimate given hydrophone geometry and the time measurement resolution of the receiver. Map was generated using R software version 4.0.3 (https://www.r-project.org/).


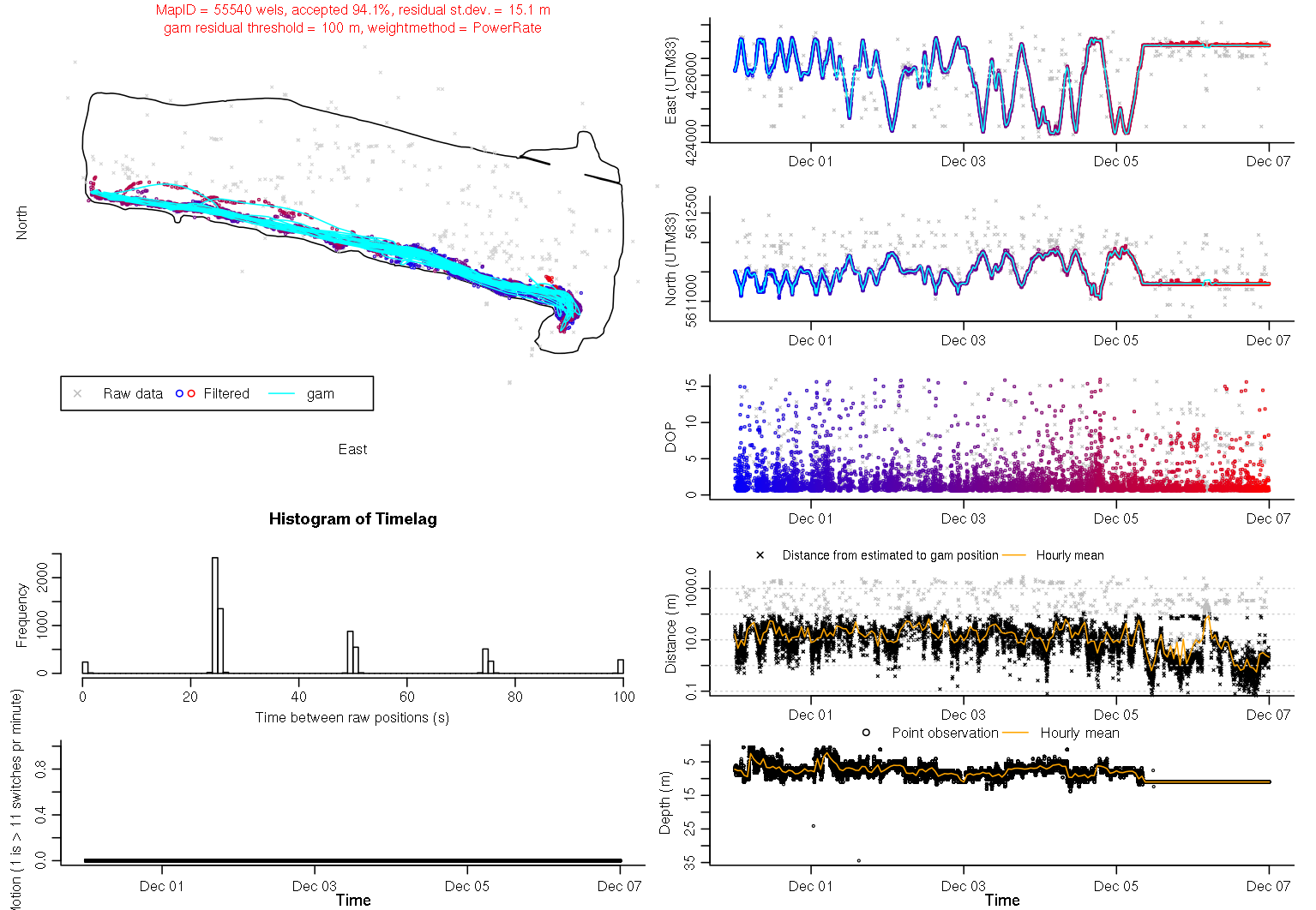


Figure S2. Example of filtering one week of position data for an European wels individual. Grey colour indicates positions not accepted by the filtering algorithm, blue to red indicates accepted positions with colour indicating time; blue being oldest and red being latest positions. The cyan-coloured line indicates the final gam-smoothed track. a) Horizontal position estimates, with the outline of Lake Milada indicated by a black line, b) easting of positions versus time, c) northing of position versus time, d) dilution of precision (DOP), a precision measure produced by U-MAP indicating the effective precision of a position estimate given hydrophone geometry and the time measurement resolution of the receiver. Map was generated using R software version 4.0.3 (https://www.r-project.org/).


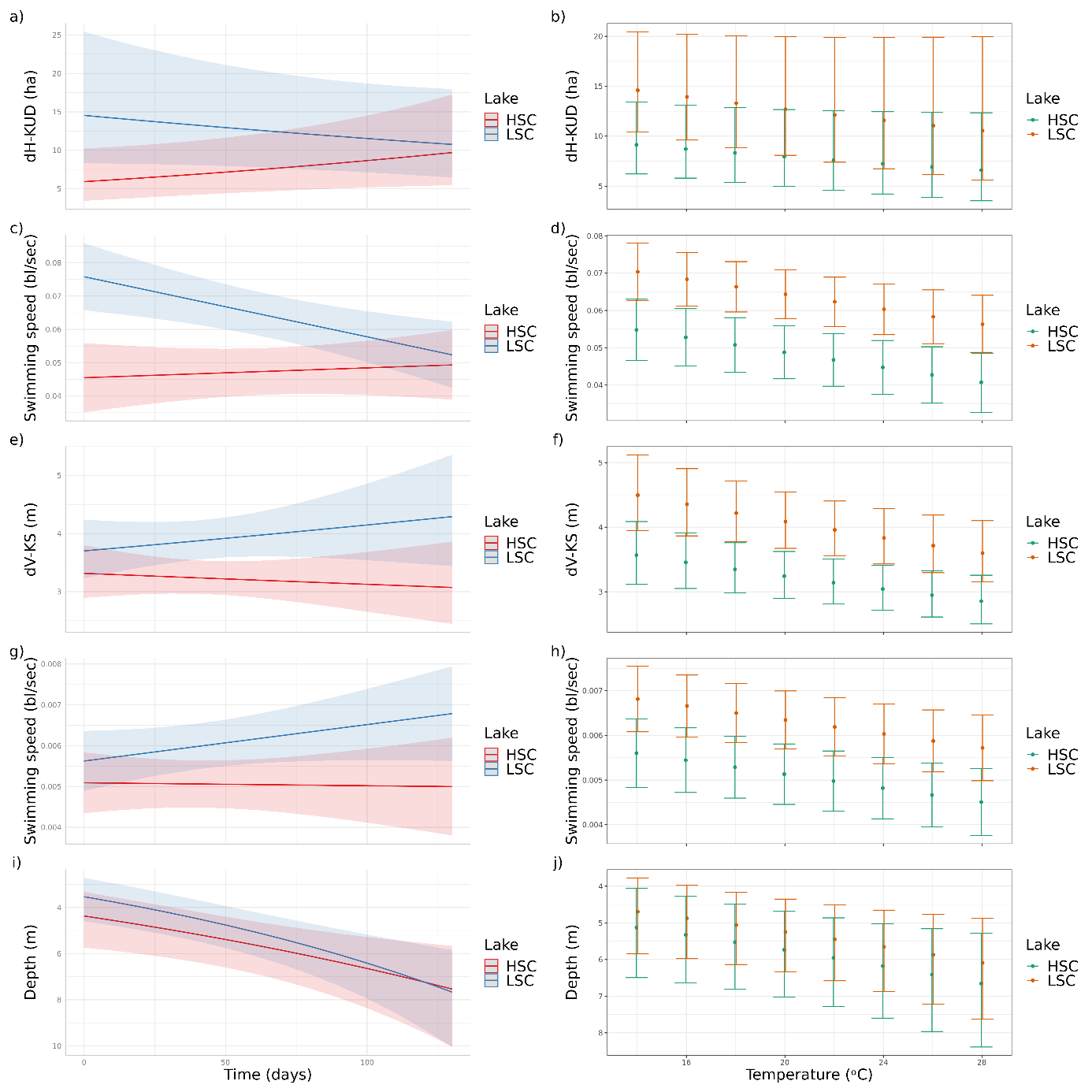


Figure S3. Model predictions of time (duration of the study) and temperature effects to extent of horizontal area (dH-KUD; a,b), horizontal activity (HA; c, d), vertical range use (dV-KS; e, f), vertical activity (VA; g, h) and mean daily depth (i, j)


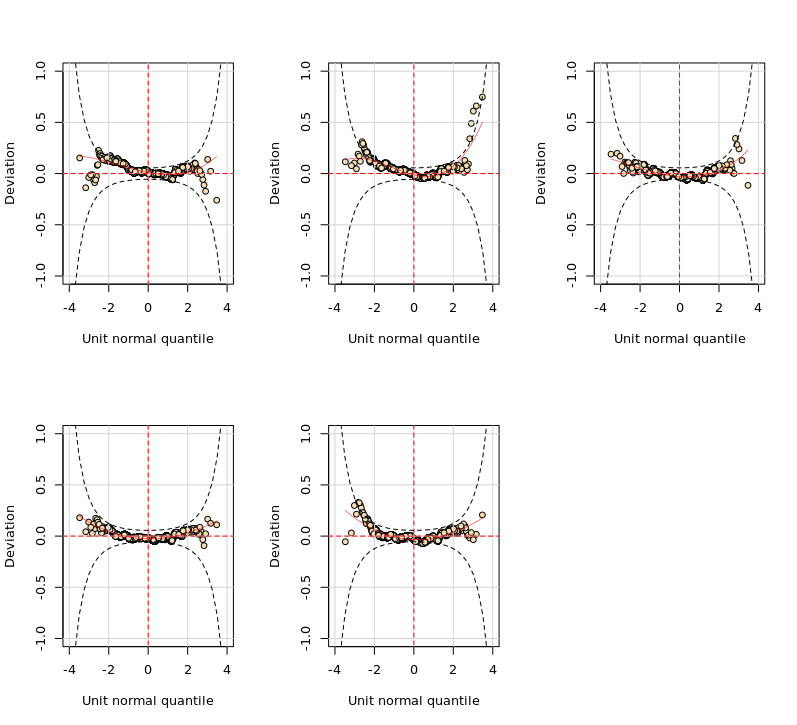
Figure S4. Worm plots of the normalized quantile residuals from the first three best BEINF(μ, σ, ν) models selected in Table B1. From up to down and left to right, models Nu1, Nu2, Nu3, Nu4 and Nu5.


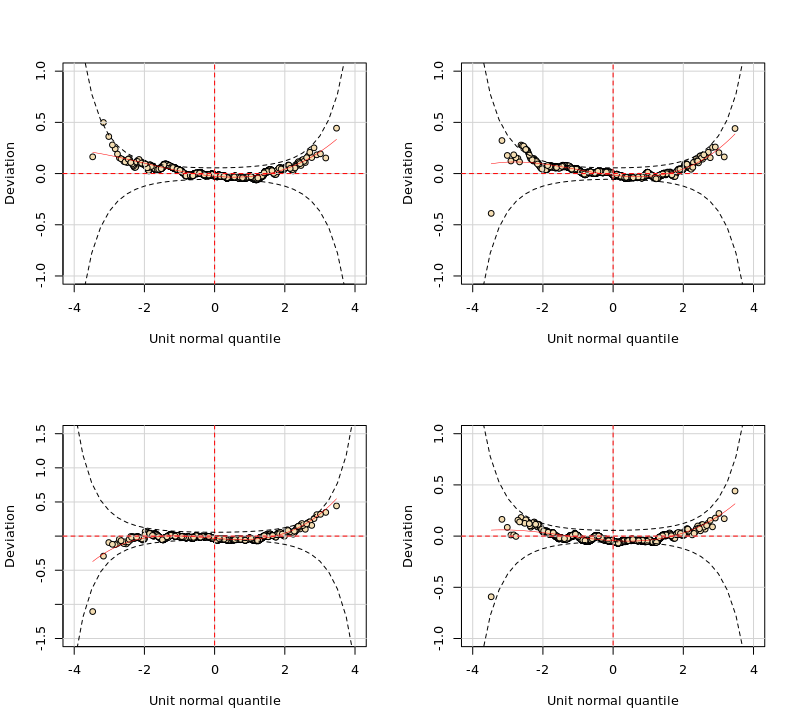


Figure S5. Worm plots of the normalized quantile residuals from the four best BEINF(μ, σ, ν, τ) models shown in Table B1, From up to down and left to right, models Tau1, Tau2, Tau3 and Tau4.


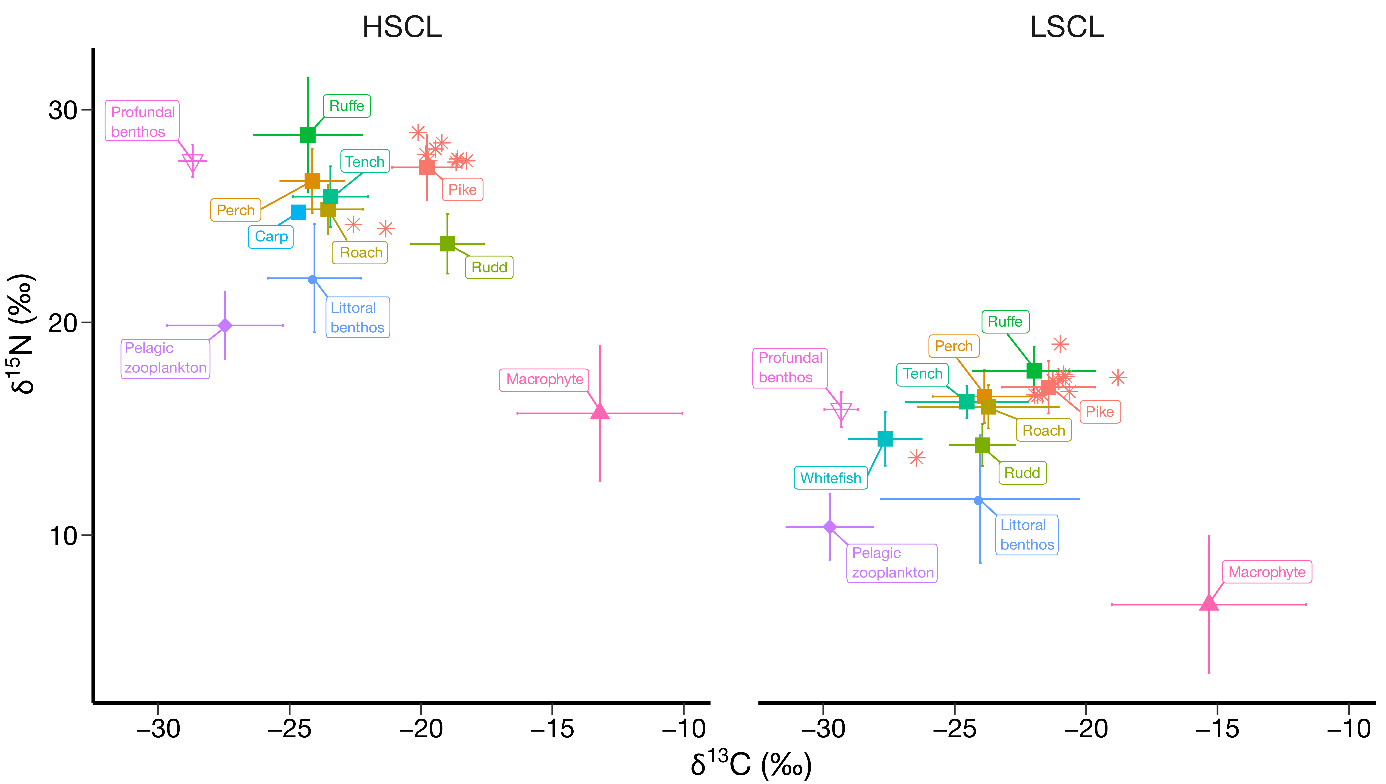


Figure S6. The mean ± SD δ13C and δ15N values of all dominant prey fish species littoral and pelagic primary producers and/or consumers collected from both study lakes in 2013-2014 (see more details in 13,14). Values of δ13C and δ15N for tracked pike individuals (asterisks) collected in the year 2015.


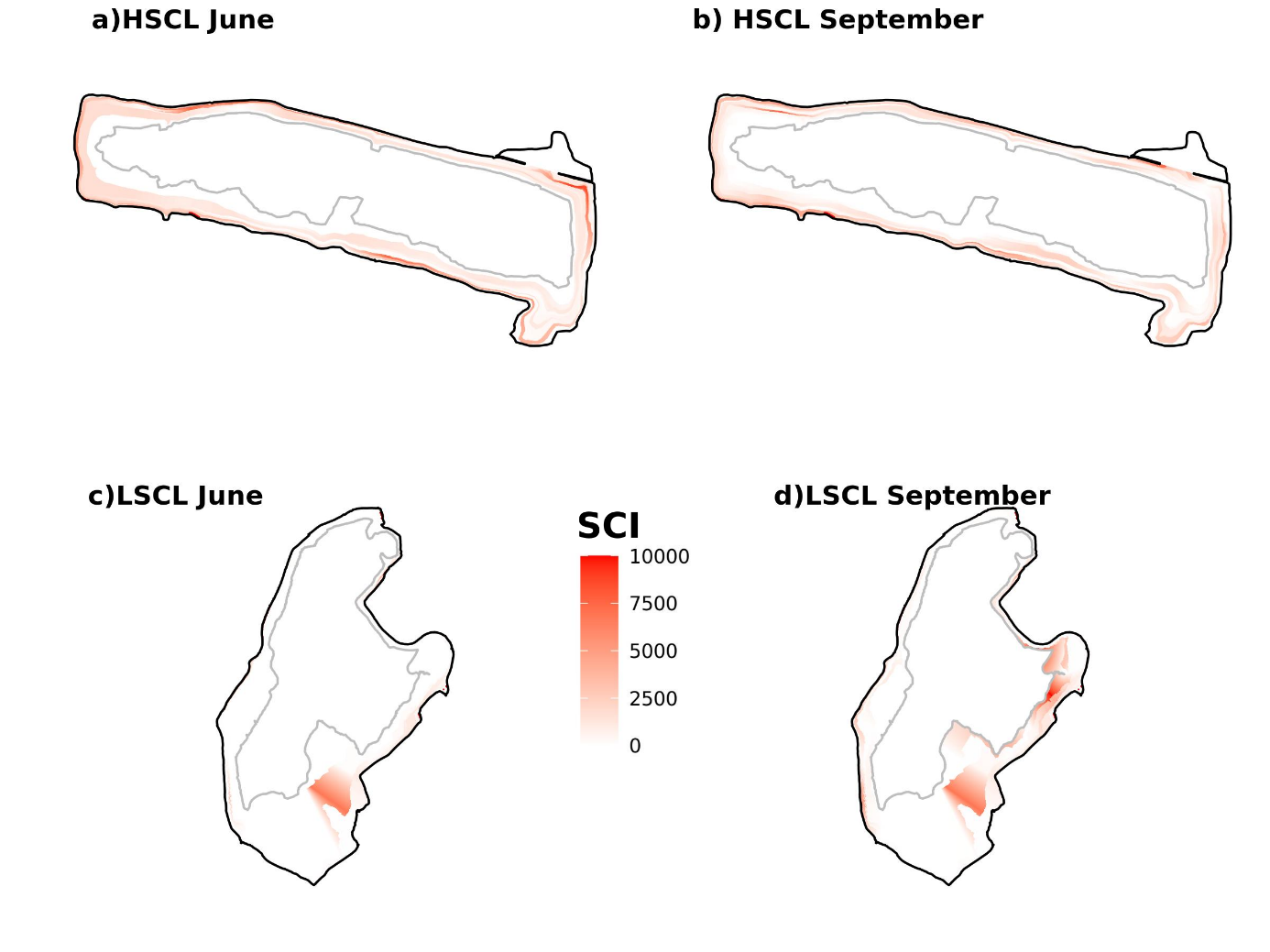


Fig. S7 Contour maps showing areal distribution of the SCI index in both lakes and structural complexity sampling sessions. Grey line display 15 m depth contour (maximal depth of macrophyte sampling). Maps were generated using R software version 4.0.3 (https://www.r-project.org/).


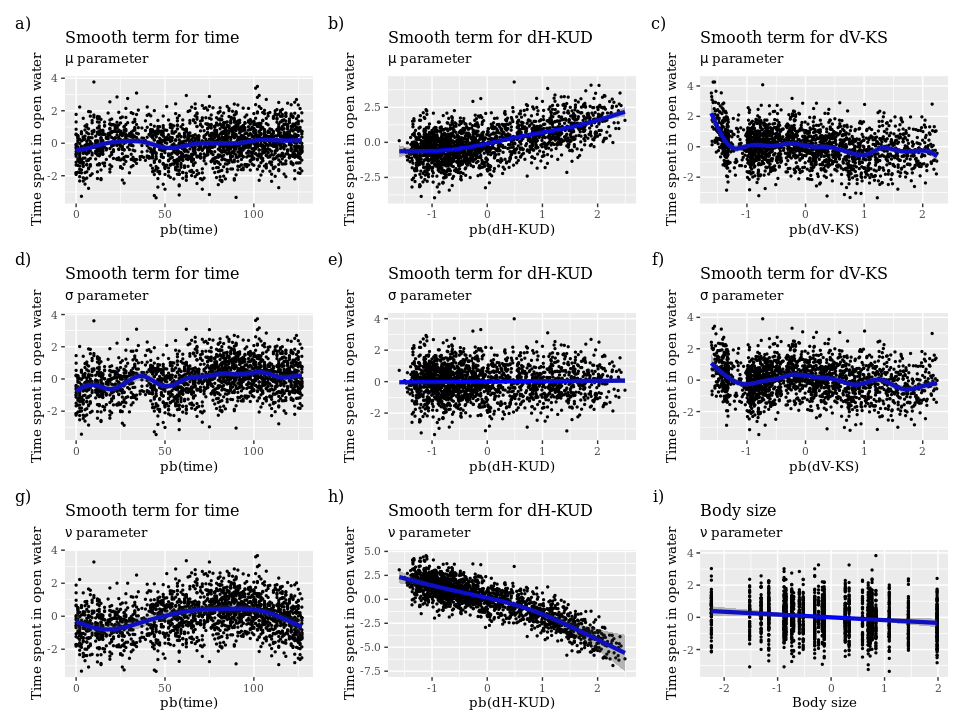


Figure S8. Relationship between the response variable and the fitted additive predictors for μ, σ and ν for a corrected (smoothing) version of the final selected model shown in Table B1 (Tau1). A smoothing P-spline function is used for the continuous explanatory variables dH-KUD, dV-KS, and time in some or all the distribution parameters. Body size is modelled as a linear predictor. The predictors at τ are omitted due to a non-significant effect (time) or non-continuous (lake) variable.
