# Supplementary material for: Contrasting structural complexity differentiate hunting strategy in an ambush apex predator
Source: Sci Rep. 2021 Sep 1;11:17472. doi: 10.1038/s41598-021-96908-1 (PMC8410764; doi:10.1038/s41598-021-96908-1)
Supplement: Supplementary file 2 — Supplementary Information 2. [file 41598_2021_96908_MOESM2_ESM.docx]

# Supplementary material 2. Positioning errors.

A total of 20 reference tags were deployed at 8 different locations during the study period (Fig. S2-1). The reference tags were Lotek model MM-M-16-50-TP, transmitting at 25 s burst interval, giving a maximum number of 144 valid positions per hour. The reference tags were suspended from a small floater and anchored to the bottom. In pelagic stations, tags were deployed at about 1, 5, and 13 m depth. In littoral stations, tags were deployed at 1 and 3 m depth. The locations were chosen to be far apart for littoral and pelagic stations, respectively, and such that the lower tag was 1 m above the bottom for pelagic stations, and 20 cm above the bottom for littoral stations. The positions of each station were taken at the surface with a GNSS device using a cm-precision RTK-service.

For each position estimate, the distance to the true position (GNSS-position) was calculated. Estimated positions were generally less than 10 m away from the true position taken at the surface, and position estimates for littoral tags were generally closer to the true position than for pelagic tags (Fig S2 -2). This difference was likely caused by larger movements in the pelagic tags, as these will shift position somewhat with changing wind direction and strength.

Positioning rates were calculated on an hourly basis for positions classified as valid, with 0 indicating no position, and 1 indicating that all transmissions resulted in a valid position estimate (Fig S2-3). Positioning rate varied over time, due to varying wind and wave conditions as well as changing temperature gradients. Positioning rate were generally higher in pelagic stations than in littoral stations, although there were considerable variation both within and between stations (Fig. S2 - 3).


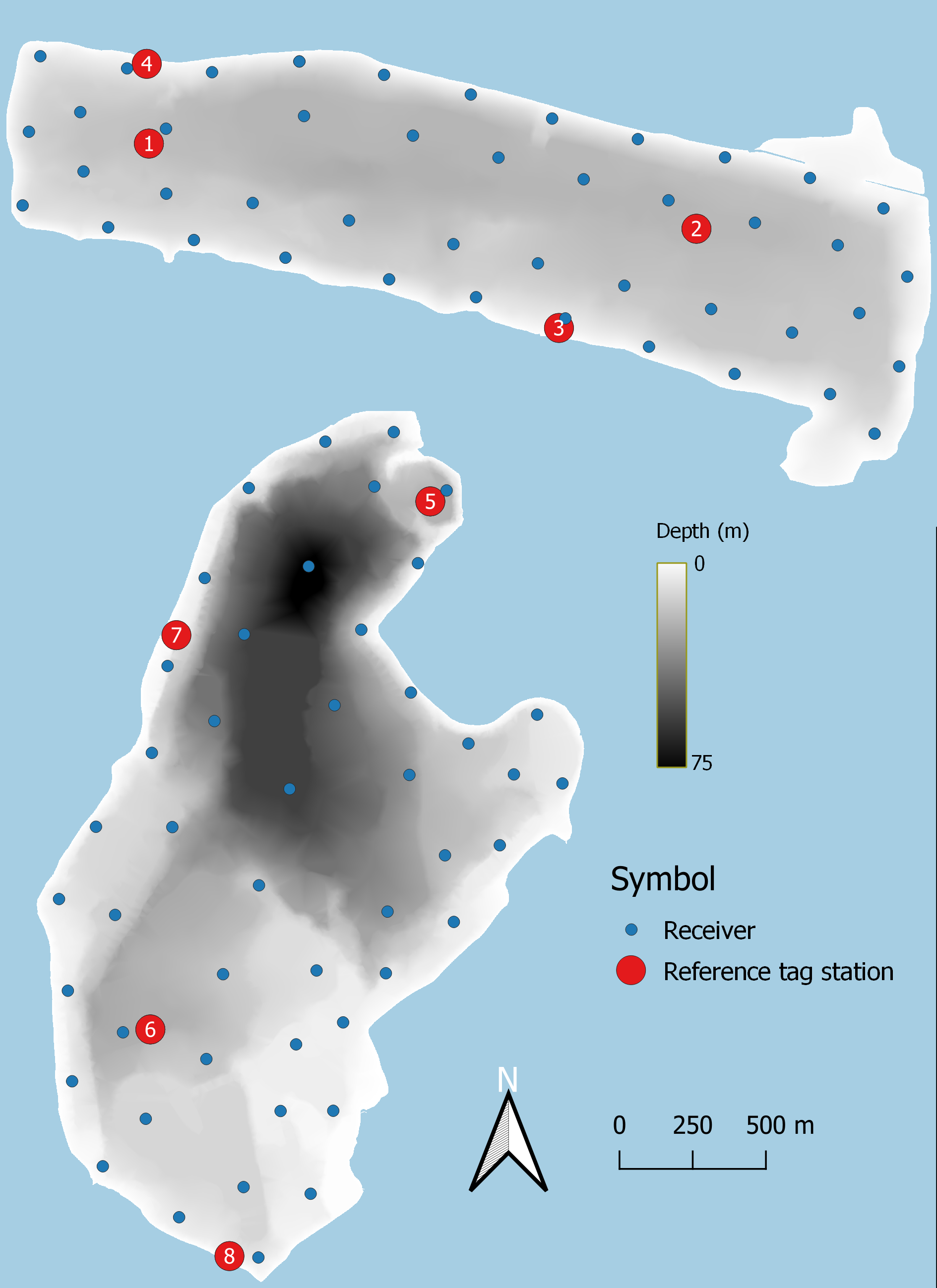


Figure S2 -1: Bathymetric maps with the location of reference tag stations. Each station had three (Station 1, 2, 5 and 6) ot two (Station 3, 4, 7, 8) reference tags at different depth, see Fig Sx2). Maps were generated using R software version 4.0.3 (https://www.r-project.org/).

Figure S2 -2: Histograms of distance between estimated position and true position for 20 refence tags throughout the study period (27 May – 10 October). Colour indicate if position was classified as TRUE of FALSE position estimate during the analysis procedure. Station locations are shown in Fig Sx1.


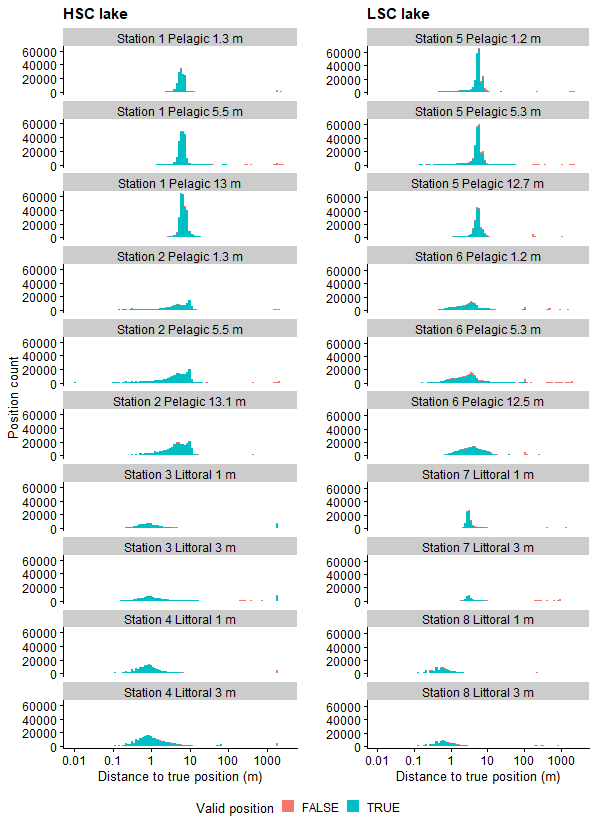


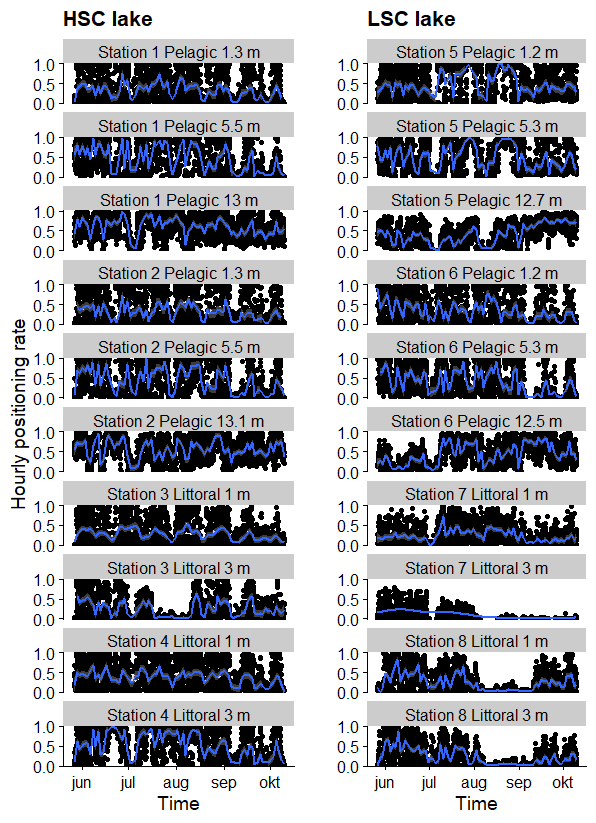
Figure S2 -3. Positioning rate for the reference tags throughout the study period. Points give hourly positioning rate observations, blue curve show results from using a gam-smoother with one knot per day. Only positions classified as valid were included in the calculations of positioning rate.
